# The SDHB Arg230His mutation causing familial paraganglioma alters glycolysis in a new *Caenorhabditis elegans* model

**DOI:** 10.1242/dmm.044925

**Published:** 2020-10-15

**Authors:** Éva Saskői, Zoltán Hujber, Gábor Nyírő, István Likó, Barbara Mátyási, Gábor Petővári, Katalin Mészáros, Attila L. Kovács, László Patthy, Shreyas Supekar, Hao Fan, Gergely Sváb, László Tretter, Arunabh Sarkar, Aamir Nazir, Anna Sebestyén, Attila Patócs, Anil Mehta, Krisztina Takács-Vellai

**Affiliations:** 1 Department of Biological Anthropology, Eötvös Lorand University, Budapest H-1117, Hungary; 2 1st Department of Pathology and Experimental Cancer Research, Semmelweis University, Budapest H-1085, Hungary; 3 HAS-SE Momentum Hereditary Endocrine Tumour Syndromes Research Group, Hungarian Academy of Sciences and Semmelweis University, Budapest H-1089, Hungary; 4 Department of Laboratory Medicine, Semmelweis University, Budapest H-1089, Hungary; 5 Department of Anatomy, Cell and Developmental Biology, Eötvös Lorand University, Budapest H-1117, Hungary; 6 Institute of Enzymology, Research Centre for Natural Sciences, Hungarian Academy of Sciences, Budapest H-1117, Hungary; 7 Bioinformatics Institute, Agency for Science, Technology and Research, 138671 Singapore; 8 Department of Medical Biochemistry, MTA-SE Laboratory for Neurobiochemistry, Semmelweis University, Budapest H-1094, Hungary; 9 Laboratory of Functional Genomics and Molecular Toxicology, Division of Toxicology, CSIR-Central Drug Research Institute, Lucknow 226031, India; 10 Division of Medical Sciences, Ninewells Hospital Medical School, University of Dundee, Dundee DD1 1NH, UK

**Keywords:** Cancer, *C. elegans*, Familial paraganglioma syndrome, TCA cycle, Succinate dehydrogenase, Warburg-like glycolysis

## Abstract

The conserved B-subunit of succinate dehydrogenase (SDH) participates in the tricarboxylic acid cycle (TCA) cycle and mitochondrial electron transport. The Arg230His mutation in SDHB causes heritable pheochromocytoma/paraganglioma (PPGL). In *Caenorhabditis* *elegans*, we generated an *in vivo* PPGL model (SDHB-1 Arg244His; equivalent to human Arg230His), which manifests delayed development, shortened lifespan, attenuated ATP production and reduced mitochondrial number. Although succinate is elevated in both missense and null *sdhb-1(gk165)* mutants, transcriptomic comparison suggests very different causal mechanisms that are supported by metabolic analysis, whereby only Arg244His (not null) worms demonstrate elevated lactate/pyruvate levels, pointing to a missense-induced, Warburg-like aberrant glycolysis. *In silico* predictions of the SDHA-B dimer structure demonstrate that Arg230His modifies the catalytic cleft despite the latter's remoteness from the mutation site. We hypothesize that the Arg230His SDHB mutation rewires metabolism, reminiscent of metabolic reprogramming in cancer. Our tractable model provides a novel tool to investigate the metastatic propensity of this familial cancer and our approach could illuminate wider SDH pathology.

This article has an associated First Person interview with the first author of the paper.

## Abstract (precis)

**Summary:** Heritable pheochromocytoma/paraganglioma follows the Arg230His missense mutation in SDHB in mitochondrial complex II. By mutating this highly conserved ortholog in *C. elegans*, we generated a tractable phenotype.

## INTRODUCTION

Rare cancers challenge biologists because of mechanistic uncertainty about their origins. Certain subtypes of neuroendocrine cancers separately described as pheochromocytomas (PHEOs) and paragangliomas (PGLs) are a case in point. Both are rare, but share histological and genetic features differing only in anatomical site of origin: those arising in the adrenal medulla are called pheochromocytomas, whereas paragangliomas are cancers of the sympathetic and parasympathetic ganglia of the peripheral nervous system, with a predeliction for the head and neck region. They are now regrouped as pheochromocytoma/paraganglioma (PPGL). The annual PPGL incidence has been estimated at 2-8 per million person per year (Pacak and Tella, 2000), but, owing to the non-specificity of the presenting symptoms, which include arterial hypertension, sweating, headache and tachycardia (Gunawardane and Grossman, 2017), the condition is almost certainly underdiagnosed (Cascon et al., 2009).

Several mutant genes have been associated with PPGL. These genes disrupt different signaling pathways (Fig. S1) and can be classified into three groups: the pseudohypoxia group [including both tricarboxylic acid cycle (TCA)- and VHL/EPAS1-related genes]; the kinase signaling group; and the Wnt signaling group. All are complemented by disease-modifying genes (Kantorovich and Pacak, 2018).

This article focuses on succinate dehydrogenase (SDH), one member of the TCA-related subgroup. This enzyme consists of four subunits – SDHA, SDHB, SDHC and SDHD – encoded by *SDHA*, *SDHB*, *SDHC* and *SDHD* genes, respectively. Further components of the TCA-related subgroup are the SDH assembly factor (SDHAF2) and the enzyme catalyzing the downstream TCA step, fumarate hydratase (FH). As the driver of mitochondrial complex II, SDH has a dual function in mitochondrial energy generation. SDH converts succinate to fumarate in the TCA cycle, but, en passant, transfers electrons into the electron transport chain via ubiquinone, by funneling electrons across the mitochondrial inner membrane towards oxygen as the final acceptor (Benn et al., 2015). The tetrameric SDH complex is membrane anchored through the SDHC and SDHD subunits. A hydrophilic flavoprotein, SDHA, inside this complex hosts the catalytic cleft of the enzyme and acts in concert with its hydrophilic partner, SDHB.

The SDHB subunit has three highly conserved iron-sulfur clusters (2Fe-2S, 4Fe-4S, 3Fe-4S) that undergo oxidation-reduction reactions as electrons pass. The two hydrophobic SDHC and SDHD partners complete the tetramer and serve as membrane anchors and the ubiquinone-binding site (Rustin and Rotig, 2002).

SDH mutations are thought to cause malignant change by three principal mechanisms. First, the sentinel event is the random allelic loss of the wild-type allele termed loss of heterozygosity (LOH). Second, it is believed that in many such cancer-linked *SDH* mutations (now renamed *SDHx*), mitochondrial succinate accumulates. This TCA intermediate can both modify proteins post-translationally and act as a signaling molecule (Murphy and O'Neill, 2018) that is released into the cytoplasm, where it can competitively inhibit oxygen-sensing prolyl hydroxylases (PHDs) (Lin et al., 2012). Following inhibition of PHD, HIF1α is not labeled for proteasomal degradation and active HIF favors pseudohypoxia-driven tumorigenesis through mal-activation of hypoxia-responsive genes such as vascular endothelial growth factor (*VEGF*; also known as *VEGFA*) or glucose transporter 1 (*GLUT1*; also known as *SLC2A1*) (Pollard et al., 2005; Selak et al., 2005). Third, it is suggested that dysfunctional SDHx can trigger increased levels of reactive oxygen species (ROS) with potential to damage macromolecules including DNA, proteins and lipids (Slane et al., 2006; Guzy et al., 2008; Tretter et al., 2016). The net result is suggested to promote tumorigenesis, but many uncertainties exist and the mechanisms have not been fully clarified.

It is curious that *SDHA*, *SDHC* and *SDHD* mutations typically cause benign tumors, whereas mutations in the gene encoding subunit B are, for unknown reasons, strongly associated with high metastatic potential. Additionally, significant numbers of SDHB-affected patients harbor germ line *SDHB* mutations and suffer from a particularly aggressive form of malignant PPGL (Amar et al., 2005; Timmers et al., 2007), whereas other family members with identical SDH mutations remain free of metastatic disease (see modifier genes above and *mev-1* below). The mechanisms are obscure. We hypothesized that a critical advance would be the establishment of a tractable animal model of an aggressive germ line form of SDHB mutation (see below). Hence, we analyzed the biological functions of the highly conserved *SDHB* homolog *sdhb-1* in the nematode *Caenorhabditis elegans*.

An extensive characterization of parts of the SDHx complex has already been performed in this nematode. The role of the *SDHC* ortholog *mev-1* is particularly well studied in response to oxidative stress (Senoo-Matsuda et al., 2001). However, little is known about worm SDHB. The *C. elegans* succinate dehydrogenase B homolog *sdhb-1* gene was first analyzed by Huang and Lemire (2009), who found a well-conserved proline residue close to the proximal quinone-binding site (Qp). When this Pro211 was mutated, which corresponds to Pro197 in the human SDHB protein, SDH assembly and function were impaired, leading to increased superoxide anion production and perturbed mitochondrial respiration (Huang and Lemire, 2009).

**Fig. 1.**
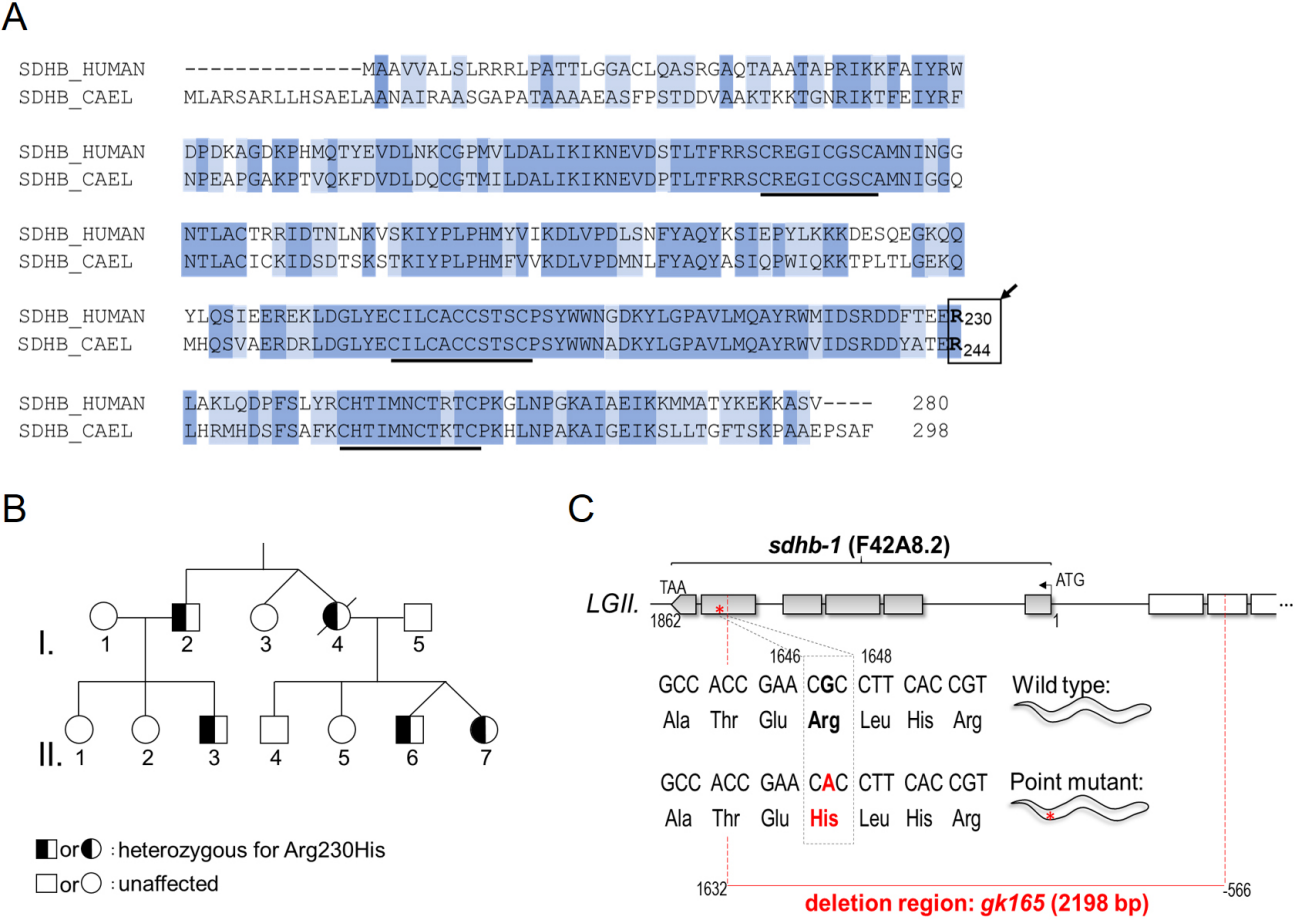
**Conservation of SDHB and its mutation in heritable** **cancer.** (A) Clustal W multiple alignment shows that *C. elegans* SDHB-1 has 60% identity and 84% similarity to the human homolog (identical or similar residues highlighted in blue and light blue, respectively). Three cysteine motifs characteristic of conserved iron-sulfur clusters are underlined. The boxed area and arrow indicate the critical Arg230 position. (B) Pedigree carrying mutated Arg230His SDHB, the mutation that can lead to pheochromocytoma/paraganglioma. Individual I.4 died of disseminated malignancy. Individuals I.2 (age 70) and I.3 (age 74) are tumor free; II.6 and II.7 (both 39, non-identical twins) each have tumors. (C) Genomic structure of the *sdhb-1* region and *gk165* deletion (dashed red lines indicate the deleted 2198 bp, including the first five exons of *sdhb-1* and the first and partially the second exon of the neighboring gene, *cup-15*). We also analyzed a transgenic strain (see Results) containing a missense mutation G1647A in the genomic sequence (G731A in the cDNA) resulting in Arg244His.

Our interest in SDHx was prompted by a family with an Arg230His missense *SDHB* mutation that leads to a familial form of malignant PHEO. This mutation has a variable penetrance estimated to be less than 50% (Hes et al., 2010). The disease first appeared in this family as a classical PHEO in the mother (see Fig. 1B for family tree), who was diagnosed in her second decade. After her death in her 50s from metastatic disease, other family members were sequenced. It was found that her non-identical twin children (twins amongst four siblings) and her own adult brother also carried Arg230His. The brother, now in his 70s, remains tumor free.

The Arg230His mutation in this family corresponds to the *C. elegans* Arg244His *SDHB-1* mutation. For readers unfamiliar with worm biology, please see Fig. S2 for the worm life cycle. First, we built nematode models of this *SDHB* mutation, where we used an existing *sdhb-1(gk165)* deletional worm and then generated two transgenic lines carrying either the above Arg244His mutation or its genomic wild-type *SDHB* as a control. Unexpectedly, our results showed that although fully deletional ‘null’ mutants arrest development soon after egg hatching at the L2 larval stage, the missense Arg244His point mutants develop much further, to the last larval L4 stage, but thereafter can only generate sterile adults. Next, we analyzed the metabolic profile of the mutants using complementary metabolic and genetic approaches, finding that Arg244His mutant animals are metabolically very different from their SDHB-null counterparts in a manner that cannot be explained simply by their differential timing of developmental arrest. The missense mutants (but not the null mutants) display aberrant glycolytic activity, principally manifesting as highly elevated lactate and pyruvate levels. Finally, bioinformatics analysis, alongside models of the SDH structure, led us to propose a pathway disorder following this missense mutation. The combined data suggest the existence of a missense-mutation-dependent, rewired form of metabolism reminiscent of the Warburg effect metabolic reprogramming that is characteristic of tumor cells. Our animal model not only provides insight into a heritable SDH impairment, but also creates a useful tool that might help to not only decipher the enigmatic nature of other *SDHx*-associated pathologies, but also shed light on the genesis of aberrant glycolysis following just one missense mutation within complex II of mitochondria.

## RESULTS

### Generation and characterization of worms carrying the *SDHB* Arg244His mutation

The *C. elegans* genome encodes a well-conserved homolog of *SDHB*, *sdhb-1 (F42A8.2)*, located on chromosome II. This SDHB-1 protein shows 60% sequence identity and 84% overall similarity to its human counterpart (Huang and Lemire, 2009) (Fig. 1A). Of note, 100% identity exists around the iron-sulphur (FeS) clusters that mediate electron transfer (Fig. 1A, underlined). In order to better understand the consequences of the familial p.Arg230His (SDHB c.689G>A), we generated the corresponding mutation, Arg244His (G731A in the cDNA) in the worm (Knudra Transgenics, Murray, UT, USA). We created two transgenic lines by inserting either the wild-type or G731A point mutant construct in single copy (at a well-defined neutral locus on the X chromosome) using transposon-mediated gene insertion (MosSCI). We made the following worm lines: COP957 [*pNU637 (sdhb-1p::sdhb-1(genomic wild-type)::sdhb-1 3′UTR; unc-119(+)) X.; unc-119(ed3) III.*] and COP952 [*pNU636 (sdhb-1p::sdhb-1(G731A)::sdhb-1 3′UTR; unc-119(+)) X.; unc-119(ed3) III.*]. These transgenic strains each contain two corresponding transgenic *sdhb-1* copies on their X chromosomes in addition to their endogenous copies on chromosome II.

To create animals carrying only two transgenic wild-type or only two G731A point mutant copies, COP957 and COP952 strains were crossed into the *sdhb-1(gk165)* null background. This deletion removes all or part of exons 1-5 of *sdhb-1* (Fig. 1C) and induces L2 larval lethality in a homozygous form (strain VC294, also known as LB713, see Huang and Lemire, 2009). This *sdhb-1(gk165)* strain can be maintained as a balanced heterozygote using the GFP-labeled balancer *mIn1mIs14*. Balancer *mIn1* is an inversion in the center of chromosome II, including *sdhb-1*, and carrying *mIs14*, which is an integrated [*myo-2::gfp; pes-10::gfp*] transgene (glowing in the pharynx of larvae and in embryos).

**Fig. 2.**
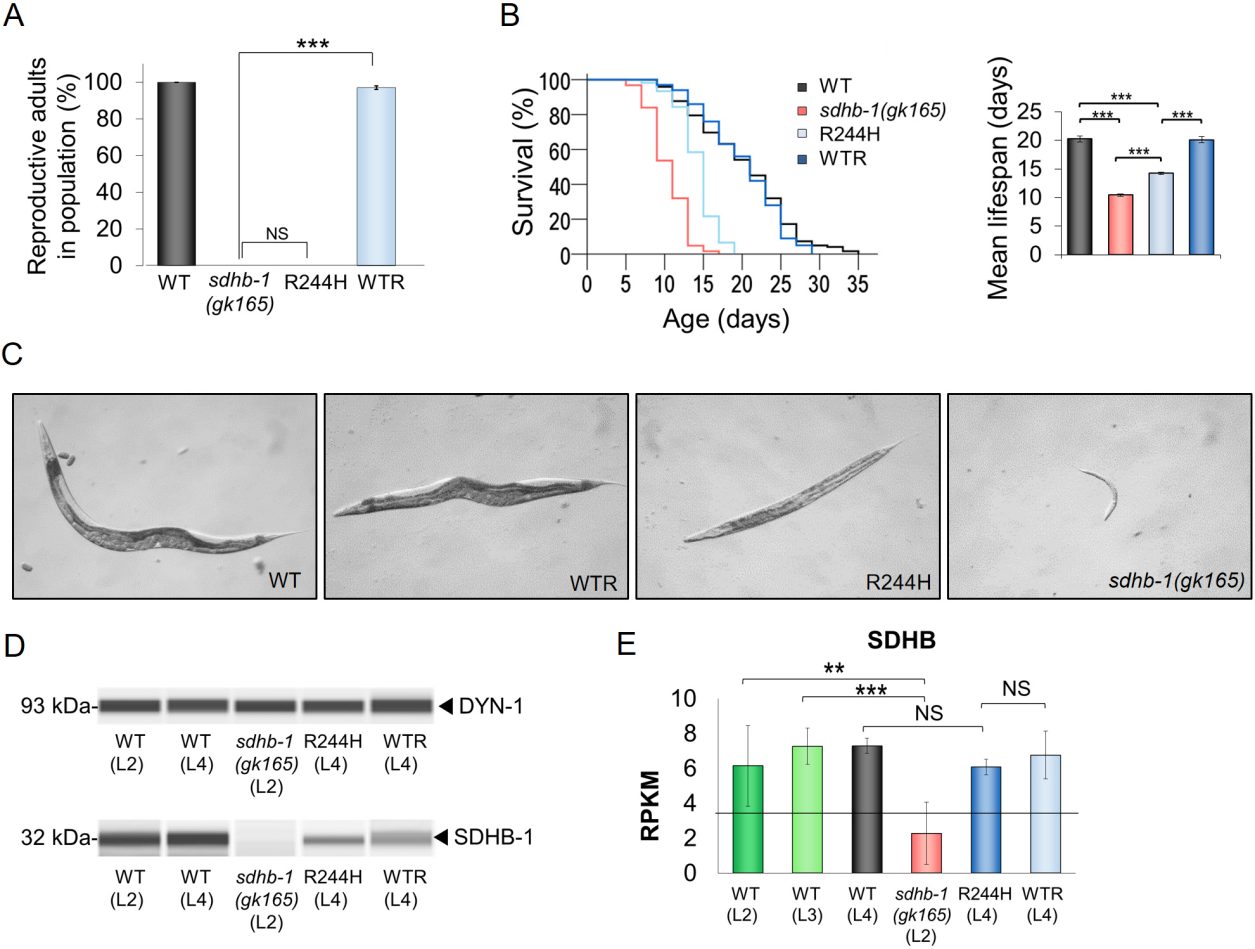
**Phenotypic analysis of nematodes carrying either null allele *gk165* or G731A missense mutation in the *sdhb-1* gene.** (A) Complementation of *gk165* null deletion by the wild-type transgene [wild type-rescued (WTR) animals, light-blue bar] led to fertile adults in 97% of the WTR cases (*n*=168) relative to wild type (WT, black bar), whereas R244H worms that reached adulthood (*n*=83) were devoid of reproductive progeny; *sdhb-1(gk165)* mutants do not progress beyond L2. (B) Lifespan of deletional and point mutant animals was measured against that of the corresponding controls. *sdhb-1(gk165)* (*n*=125) mutants and R244H (*n*=166) transgenic animals showed a significantly shorter survival compared to their respective controls WT (*n*=241) and WTR (*n*=100) (*n*=3 separate experiments). (C) At 20°C, on day 3 after hatching, WT and WTR worms grew into fertile adults, while R244H animals reached adulthood only on day 4-5, suggestive of delayed development. The *sdhb-1(gk165)* animals showed arrested development at the L2 larval stage. (D) Wes experiment shows that the 32 kDa band specific for SDHB-1 is absent in *gk165* null mutants, but is present in all control strains (WT and WTR). R244H animals also express SDHB-1 [relative to DYN-1 (93 kDa) loading control]. (E) RNA-seq data related to *sdhb-1* also verify Wes results: *sdhb-1* message is present in all examined strains except *gk165* null mutants, for which the *sdhb-1*-specific mRNA lies below the detectable limit. RPKM, reads per kilobase per million mapped reads. The black horizontal line represents a threshold, below which no functional protein is observed. Statistical significance is indicated in each graph as ***P≤0.001 and **P≤0.01 versus the corresponding sample (NS, not significant; independent two-sample Student's *t*-test with Bonferroni correction). Error bars represent s.e.

First, we crossed wild-type (COP957) and G731A point mutant (COP952) transgenic lines into *sdhb-1(gk165)* null mutant background and determined whether these transgenes were able to rescue the deletion caused by *gk165.* As a validation of our method, and using over 200 worms, we first observed that only the wild-type transgene could complement the deletion [rescue in 97% of worms, which we will refer to hereafter as wild type rescued (WTR); Fig. 2A; Fig. S3)]. In contrast, the G731A rescue generated sterile adults with a protruding vulva (the ‘Pvl’ phenotype; Fig. S3); these phenotypes were not further analyzed. Henceforth, we refer to the G731A point mutant animals on a deletional background as R244H (Arg244His) (Fig. 2A; Fig. S3). To facilitate propagation, the R244H animals were also balanced using *mIn1mIs14*, as for *gk165* deletional mutants.

Next, we compared the lifespan of homozygous *sdhb-1(gk165)* mutants against wild-type worms and observed that *gk165* homozygotes survived about half as long as wild-type control N2 (WT) animals (Fig. 2B; Table S1), consistent with the data of Pujol et al. (2013), who found that *sdhb-1*-specific RNA interference (RNAi) treatment also resulted in a similarly shortened lifespan. R244H worms also showed a shortened lifespan compared to WT and WTR animals (Fig. 2B; Table S1). In addition, we examined the effect of the above mutations on development. At 20°C on day 3 after hatching, WTR generated viable adults similar to WT worms, whereas *sdhb-1(gk165)* animals showed arrested development at L2, as expected. In contrast, R244H worms reached adulthood on only day 4-5, indicating delayed development (Fig. 2C).

In order to investigate the proteins generated by the *gk165* and Arg244His *sdhb-1* mutant alleles, we performed Western capillary immunoassay (Wes) experiments using a human SDHB-specific antibody, which successfully cross-reacted with the worm SDHB-1 protein, likely due to the high level of conservation. As expected, *gk165* represents a null allele as no antibody specific band can be detected, whereas the same antibody recognized a single, well-defined band of 32 kDa, specific for SDHB-1 in all control samples (Fig. 2D). In R244H mutants, the point mutant protein is also detectable, but present in a lower quantity (Fig. 2D). Transcriptomics data verify Wes results at the mRNA level: we find that *sdhb-1* mRNA lies below the detectable limit in *gk165* mutants, whereas in R244H point mutants, *sdhb-1* mRNA is transcribed, although at a decreased level (Fig. 2E; Table S8).

**Fig. 3.**
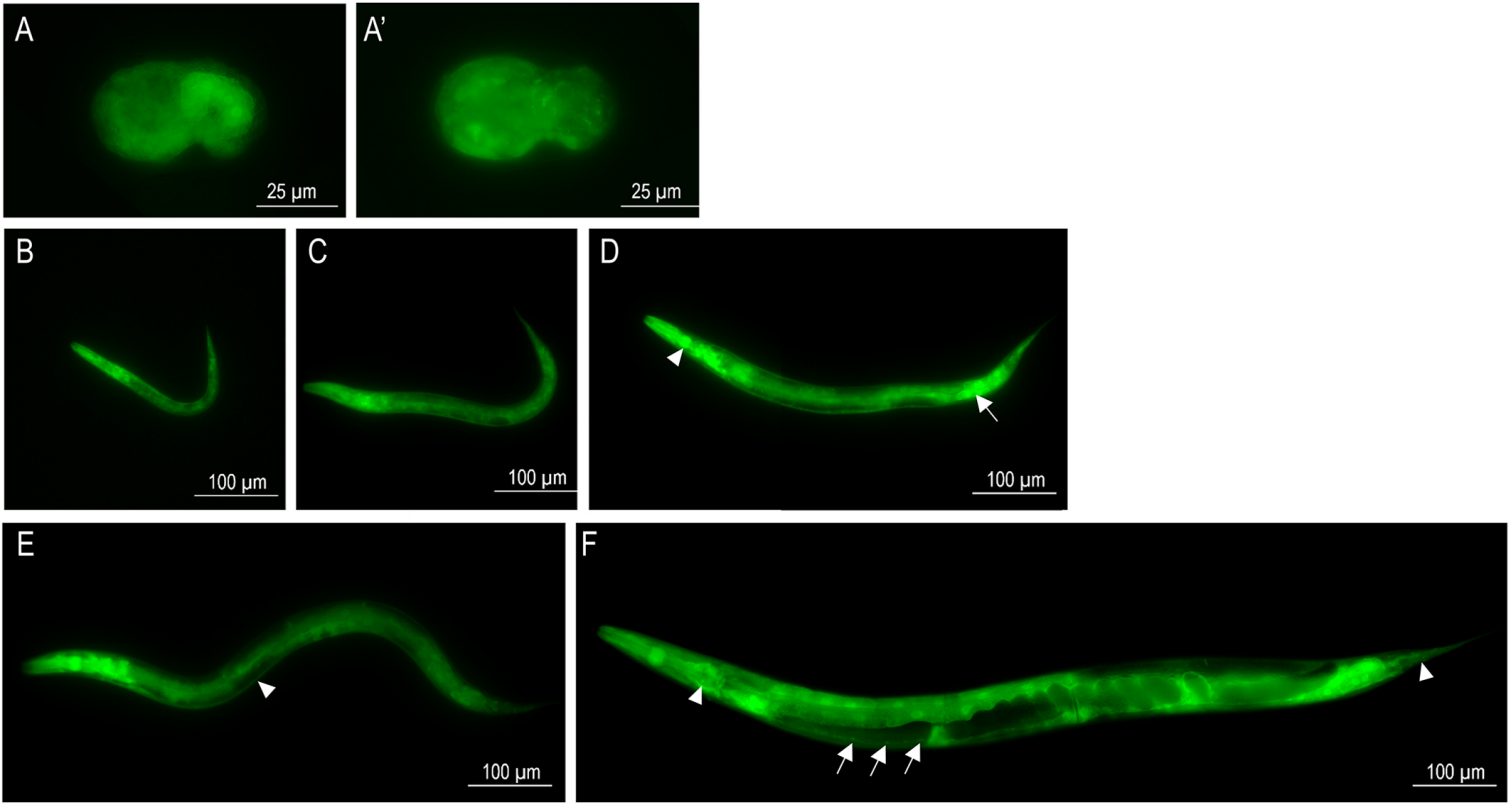
**SDHB-1 is ubiquitously expressed throughout worm development.** (A,A′) A transcriptional reporter construct shows strong expression of SDHB-1 in embryos in the comma stage (A, lateral view; A′, ventral view). (B-F) SDHB-1 expression persists throughout larval stages (B, L1; C, L2; D, L3; E, L4) into adulthood (F) with intense expression in the pharynx (D, arrowhead), together with intestinal (D, arrow) and hypodermal (E, arrowhead) cells, head and tail neurons (F, arrowheads) and the ventral nerve cord (F, arrows). In all panels, anterior is to the left and dorsal is up. These panels are composites.

In addition, we generated a *psdhb-1::gfp* strain carrying an extrachromosomal array of a transcriptional reporter construct and analyzed its expression pattern. We found that the transgene begins to be expressed from the comma stage during embryogenesis (Fig. 3A,A′) and persists throughout larval stages (Fig. 3B-E) into adulthood (Fig. 3F). SDHB-1 is expressed in the pharynx (Fig. 3D), intestinal (Fig. 3D) and hypodermal (Fig. 3E) cells, head and tail neurons (Fig. 3F), and in the ventral nerve cord (Fig. 3F).

**Fig. 4.**
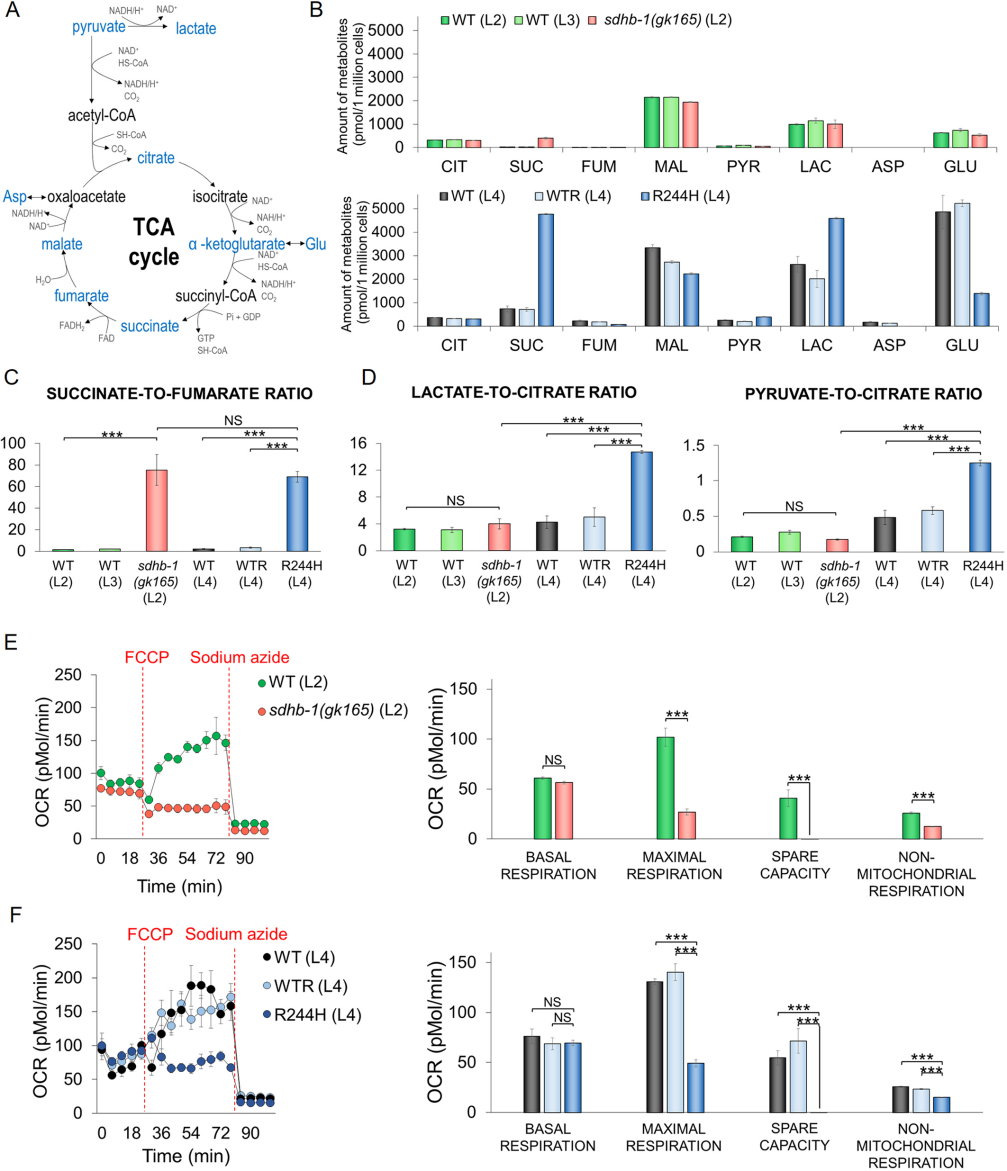
**TCA cycle and related metabolites in *sdhb-1* null and R244H point mutant worms and their effects on oxygen consumption.** (A) TCA cycle and related metabolites: metabolites (blue) were analyzed by LC-MS in WT L2 (*n*=2300), L3 (*n*=2000), L4 (*n*=1750) larvae, *sdhb-1(gk165)* null mutants (*n*=2300), WTR (*n*=1750) and R244H point mutant (*n*=1750) animals. (B) Results from *n*=6 separate experiments measuring citrate (CIT), succinate (SUC), fumarate (FUM), malate (MAL), pyruvate (PYR), lactate (LAC) and glutamate (GLU) in deletional mutants and their developmental-stage control L2 and L3 WT animals (charts in red, dark green and light green, respectively) or in R244H mutants and their control L4 WTR and WT animals (charts in dark blue, black and light blue, respectively). Aspartate (ASP) was detected only in L4 WT and WTR animals (undetectable in L4 R244H point mutants). Alpha-ketoglutarate levels were undetectable in all (not shown). (C) The succinate-to-fumarate ratio was increased equivalently in deletional and point mutants. (D) The metabolic profile of the R244H mutant differed from that of the controls. Lactate-to-citrate and pyruvate-to-citrate ratios were calculated based on similar citrate levels in each worm type. (E,F) The effect of the deletion versus point mutation on the oxygen consumption (OCR) by Seahorse technique (*n*=8 parallel experiments). (E) In the case of *sdhb-1(gk165)* mutants (*n*=800), oxygen consumption was not increased after FCCP treatment compared to wild-type control. (F) R244H point mutants (*n*=260) failed to show accelerated oxygen consumption in response to FCCP, as also seen in the null worms. Statistical significance is indicated in each graph as ***P≤0.001 versus the corresponding sample (NS, not significant; one-way ANOVA). Error bars represent s.e.

Metabolic characterization of *sdhb-1* deletional and *SDHB* Arg244His point mutants shows elevated succinate-to-fumarate ratios and attenuated oxygen consumption with reduced mitochondrial and ATP content. Deficiency of SDH impairs the conversion of succinate to fumarate, thereby disrupting the TCA cycle (Fig. 4A). Therefore, we compared TCA-related metabolite levels, glycolytic activity and the efficiency of stimulated mitochondrial oxygen consumption in *sdhb-1(gk165)* null mutants, R244H point mutants, WTR worms (which served as comparators for point mutants) and wild-type L2, L3 and L4 larvae as further stage-dependent controls.

Metabolite concentrations (succinate, fumarate, malate, citrate, glutamate, alpha-ketoglutarate, aspartate, pyruvate and lactate) were measured by liquid chromatography–mass spectrometry (LC-MS) (Fig. 4B; Table S2) using methods described earlier (Hujber et al., 2018), but adapted herein, as follows. In preliminary experiments, extracts from multiple independent worm batches determined the lower limit of detection of the above metabolites (five independent batches; data not shown). Next, individual worms of a given genotype and larval stage were picked manually. Finally, worm number was normalized to a common cell number to compare metabolite data from different genotypes, shown as pmol/million cells (Fig. 4B; Table S2).

Citrate and malate levels were similar across strains, providing a ready reference measurement when different metabolites were compared in the examined genotypes. The succinate precursor, alpha-ketoglutarate (aKG), level was always below the detection limit, irrespective of genotype. The SDHB product, fumarate, was present in small quantities. As expected, succinate was elevated in the *sdhb-1(gk165)* deletional mutant, but, interestingly, R244H point mutants also showed an equivalent succinate rise (Fig. 4B; Table S2). The succinate-to-fumarate ratio was similarly elevated in both mutants despite their different developmental stages (Fig. 4C). These data pointed to a complex metabolic phenotype dependent on the mutation status of SDHB. For example, both aspartate and glutamate levels were decreased in R244H point mutants compared to wild-type and transgenic WTR L4 animals (Fig. 4B; Table S2). Furthermore, R244H point mutants (but not null mutants) displayed significantly higher pyruvate and lactate levels compared to their larval stage-matched L4 controls (Fig. 4D; Table S3).

Because the SDH complex plays a parallel role in complex II of the mitochondrial respiratory chain (see Introduction), we next determined the stimulated mitochondrial oxygen consumption rate (OCR) using the Seahorse technique in the above *sdhb-1* deletional worms, point mutants and control strains.

The Seahorse XF96 Metabolic Analyzer has previously been used in whole organisms such as *C. elegans* (Luz et al., 2015; Koopman et al., 2016). We used this device to study the action of two mitochondrially active compounds, protonophore carbonyl cyanide p-(trifluoromethoxy) phenylhydrazone (FCCP) and the complex IV (cytochrome oxidase) inhibitor sodium azide, to assess stimulated bioenergetics in *sdhb-1* mutant worms and control strains (Fig. 4E,F). There were no differences in either the unstimulated, basal OCR or the OCR following sodium azide addition between the mutants and control animals. The addition of protonophore FCCP resulted in elevated, maximal oxygen consumption in all control animals. In marked contrast, in *gk165* deletional and R244H mutants, oxygen consumption was unchanged after FCCP injection, implying a loss of reserve capacity of respiration in these mutants (Fig. 4E,F; Table S4).

**Fig. 5.**
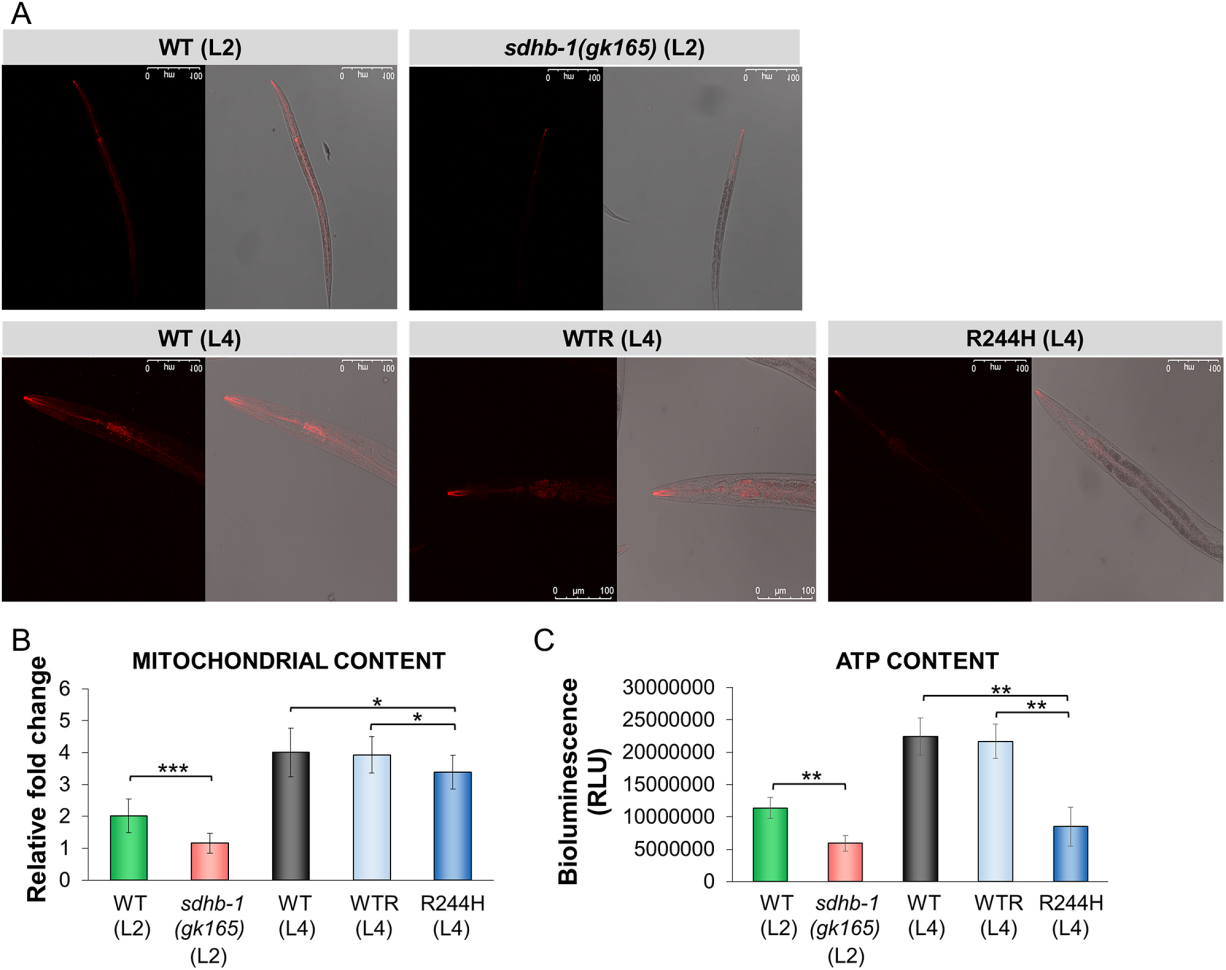
***sdhb-1* mutants show reduced ATP production and functional mitochondrial content.** (A) Confocal microscope images (40×) showing comparative mitochondrial accumulation of Mitotracker Red CMX Ros dye showing reduced fluorescence in *gk165* null mutants and R244H point mutants versus respective rescue or control strains. (B) Relative fold change of the mitochondrial content in the above mutants versus stage-specific controls (*n*=10). (C) ATP content analyzed by ATP Bioluminescent Assay (*n*=2000) as relative light units (RLU). Statistical significance is indicated in each graph as ***P≤0.001, **P≤0.01 and *P≤0.05 versus the corresponding sample (independent two-sample Student's *t*-tests with Bonferroni correction). Error bars represent s.e.

As the SDH complex is an integral part of complex II of the mitochondrial electron transport system and its deficiency may hamper mitochondrial integrity and hence energy production, we next estimated the functional mitochondrial content using Mitotracker dye, the uptake of which is dependent on mitochondrial transmembrane potential and measured total ATP production, using a bioluminescent ATP assay. We observed that deletional as well as point mutants of *sdhb-1* had not only significantly fewer healthy mitochondria but also demonstrated downregulated ATP production, compared to wild-type and rescued strains (Fig. 5; Tables S5 and S6).

### Possible impact of the Arg244His mutation on the molecular structure of SDHB-1 in *C. elegans*

**Fig. 6.**
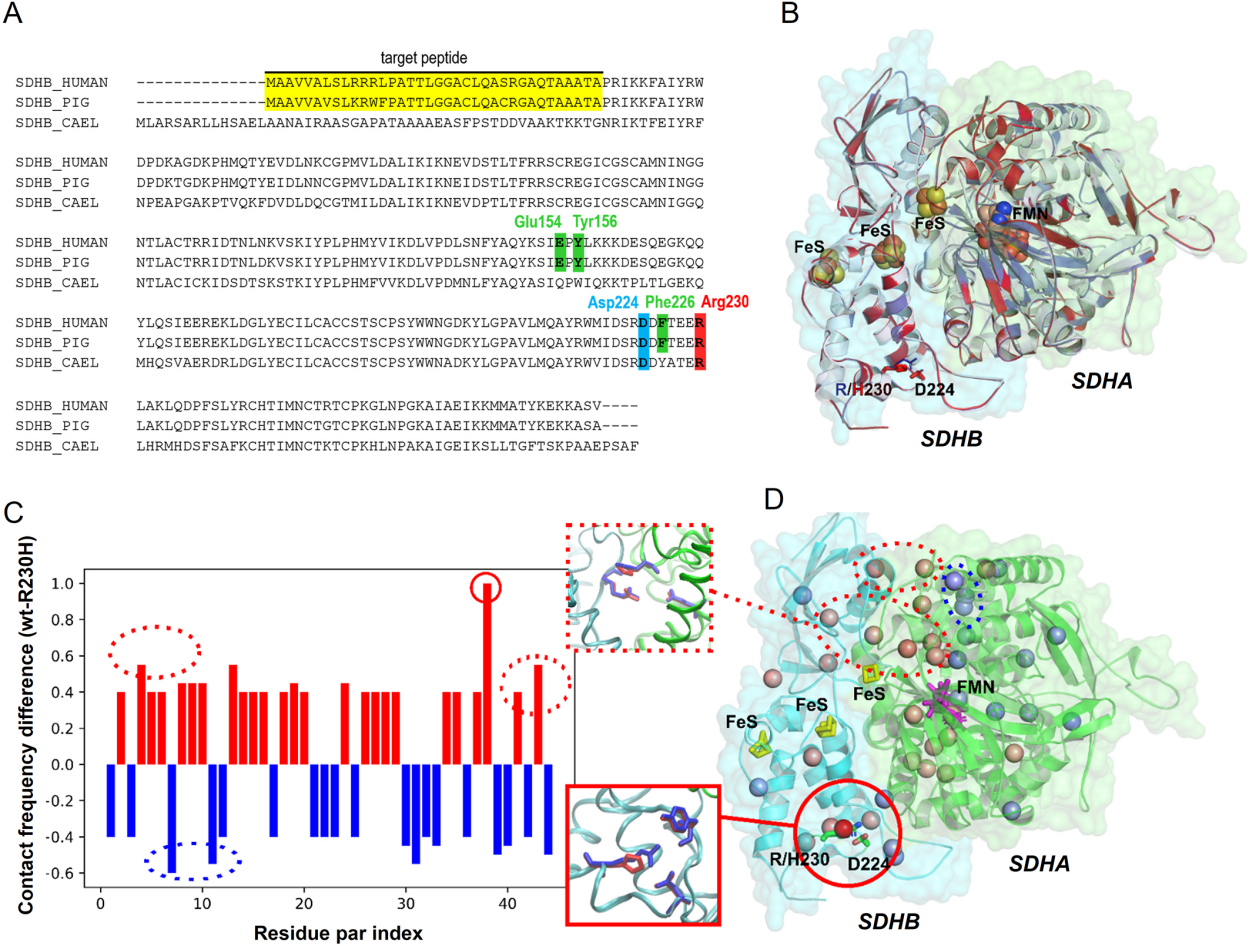
**The effect of the Arg230His point mutation in the SDHB subunit on the structure and function of the SDHA/B enzyme complex.** (A) Sequence alignment of SDHB sequences. Asp224 and Arg230, which form a salt bridge in porcine SDHB, are highly conserved, and marked in the alignment in blue and red, respectively. The positions of residues Glu154, Tyr156 and Phe226, the peptide backbone atoms of which also form hydrogen bonds with Arg230, are marked in green in the alignment. (B) Superimposition of the top-scoring wild-type (blue) and R230H (red) human SDHA/B homology models to porcine SDHA/B X-ray structure (PDB ID 4YTP; shown in white). Co-factors are shown in sphere representation, while residues 224 and 230 of SDHB are shown in stick representation for the wild-type and mutant models in blue and red, respectively. (C) Differences in averaged residue-pair contact frequencies from the 20 top-scoring models. This plot illustrates residue pairs that exhibit a loss (positive) or gain (negative) of contacts in more than 40% (>8 out of the 20 models) going from wild-type to the R230H mutant. (D) Structural annotation of the major contact changes in SDHA/B complex upon R230H mutation as seen in C (>40%). The beads in the inter-residue space indicate the location and the extent of structural perturbation. The extent of perturbation upon mutation is color coded in RWB space [R=1; W=0; B=–1], with red indicating loss of contact and blue indicating formation of a new contact. Highest perturbation is seen near the mutation site (see solid red line inset), but a significant loss of contacts is also observed at the interface of SDHA and SDHB (see dotted red line inset). FeS, iron-sulphur; FMN, flavin mononucleotide.

The Arg244His mutation of *C. elegans* SDHB-1 corresponds to the Arg230His mutation of human SDHB, which leads to a familial form of malignant PPGL (Brouwers et al., 2006; Cerecer-Gil et al., 2010). We hypothesized, from the conservation of residues around this site (indicated by the boxed area and arrow in Fig. 1A), that Arg230 plays an equivalent role across the SDHBs of these distant species. In the X-ray structure (PDBID 4YTP) of the SDHB subunit of porcine heart (Inaoka et al., 2015), the guanidino group of Arg230 of SDHB forms two hydrogen bonds with the carboxyl oxygens of the side chain of Asp224. In addition, Arg230 is hydrogen bonded with the peptide backbone atoms of residues Glu154, Tyr156 and Phe226. The structural importance of the Asp224-Arg230 salt bridge is supported by the observation that both residues are conserved from bacteria to mammals (Fig. 6A). Hence, substitution of Arg230 by other residues, such as His, Cys, Gly and Leu (Cerecer-Gil et al., 2010), is likely to disrupt the strong hydrogen bonding between residues 224 and 230, which might impair protein function (see Discussion). In order to probe the effect of R230H mutation in human SDHB, we constructed homology models of the human SDHA/B complex for the wild type and R230H mutant, based on the above structure of the porcine SDHB subunit (Fig. 6B). To evaluate the structural changes induced by the mutation, we calculated all pairwise residue contacts for the 20 top-scoring wild-type and mutant models. The difference in averaged pairwise contacts from wild type to R230H mutant indicates a reduction of about five inter-residue contacts upon the mutation (Fig. 6C,D; see also Table S7 and Fig. S4 for a detailed description and a network representation of the sidechain perturbations seen in the models as a result of mutation). Although the most prominent perturbations are close to the mutation site, we also find an appreciable loss of contacts at the interface between the distant N-terminal domain of SDHB (∼30 Å away from the mutation site) and SDHA (Fig. 6D; Fig. S4). The structural models suggest that the reduction of inter-residue contacts, along with disruption of the SDHA-SDHB interface, might render the complex inactive or, alternatively, result in aberrant SDH activity after the distal R230H mutation.

### Transcriptomic analysis reveals a metabolic shift towards glycolytic activity in Arg244His point mutants

We used a transcriptomic analysis of RNA extracted from deletional and point mutants versus stage-specific controls to better understand our observed phenotypic differences. We found that expression of genes encoding vitellogenins (such as *vit-2*, *vit-5*, *vit-6*) and genes required to build the cuticle (for example, *col-140*, *col-122*, *col-123* or *bli-6*) were increased in the point mutant worms, consistent with their later developmental arrest point (point mutants develop to adulthood whereas null mutants arrest at L2). We observed that R244H point mutants also show a decreased expression of germline-specific genes (*gld-1* or *spch-1*) when compared to wild-type worms, which might relate to the sterility of R244H animals. Interestingly, given the neurological nature of the disease, in deletional mutants, some genes associated with the nervous system (for example, *C06B3.6*, *B0205.13*, *F18E3.12*, *F09F7.6*) are highly overexpressed, which requires further study (data not shown).

**Fig. 7.**
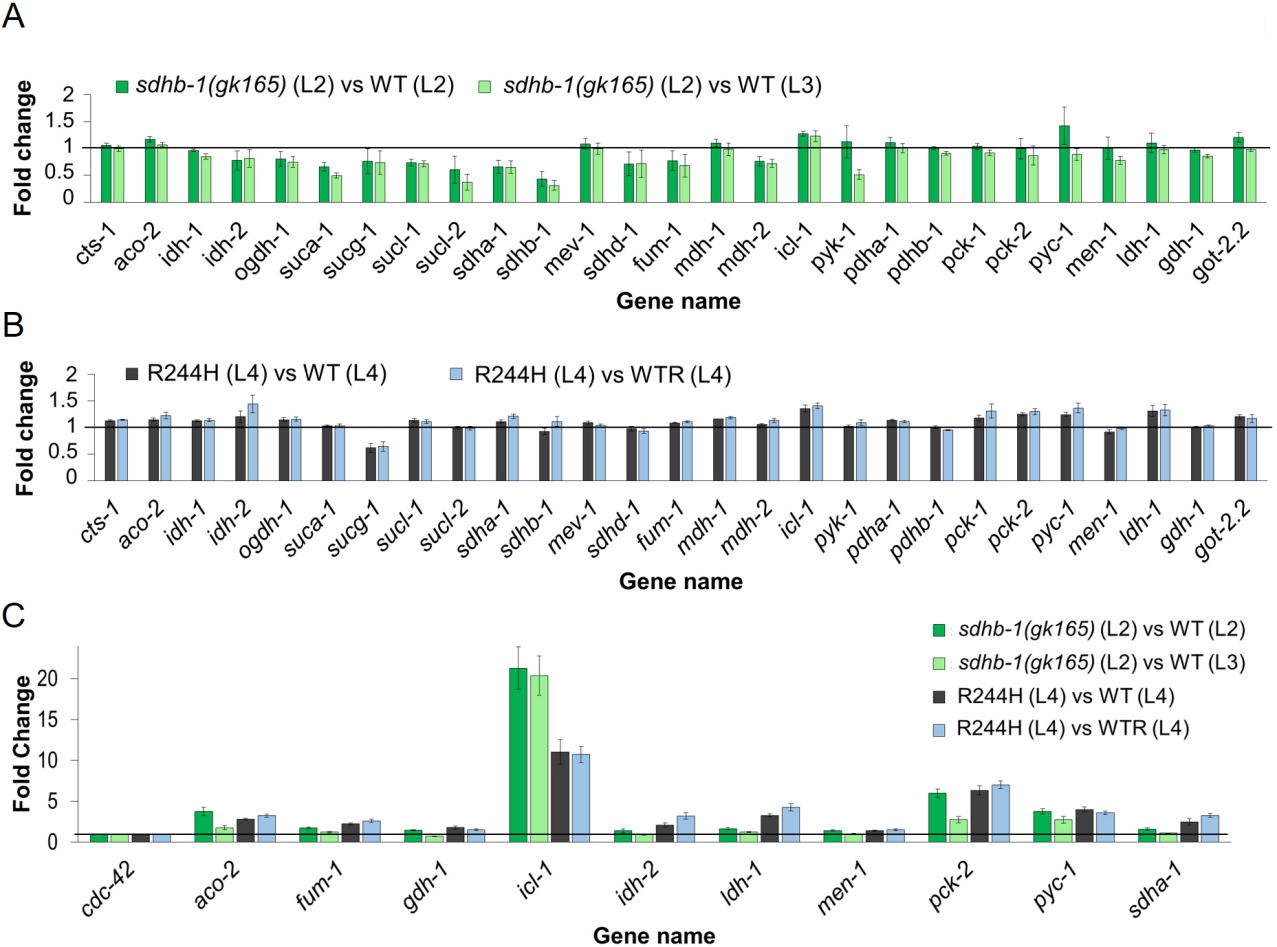
**Transcriptomic analysis of *sdhb-1(gk165)* deletional and Arg244His point mutants reveals different metabolic alterations.** (A,B) Transcriptomic analysis in *sdhb-1(gk165)* deletional mutants (*n*=5000) (A) and R244H (Arg244His) point mutants (*n*=2500) (B), relative to their wild-type stage controls. (C) qPCR experiments performed for a subset of genes analyzed by transcriptomics in *sdhb-1(gk165)* deletional and R244H (Arg244His) point mutants. In A-C, dark-green and light-green columns show the ratio of mutant expression versus WT L2 (*n*=5000) and L3 (*n*=5000) controls, respectively. Black and blue columns show the ratio of mutant expression versus WT L4 (*n*=2500) and WTR L4 (*n*=2500) controls, respectively. The genes analyzed are indicated in the *x*-axes; fold change is represented in the *y*-axes. Underexpressed genes have values between 0 and 1; overexpressed genes have values from 1 upwards. Genes encoding the following enzymes were analyzed: CTS-1, citrate synthase; ACO-2, aconitase; IDH-1/2, isocitrate dehydrogenase; OGDH-1, oxoglutarate dehydrogenase; SUCA-1, succinyl-CoA ligase (ATP-specific) beta subunit; SUCG-1, succinyl-CoA ligase (GTP-specific) beta subunit; SUCL-1/2, succinyl-CoA ligase (ATP/GTP-specific) alpha subunit; SDHA-1, succinate dehydrogenase A subunit; SDHB-1, succinate dehydrogenase B subunit; MEV-1, succinate dehydrogenase C subunit; SDHD-1, succinate dehydrogenase D subunit; FUM-1, fumarase; MDH-1/2, malate dehydrogenase; ICL-1, isocitrate lyase/malate dehydrogenase; PYK-1, pyruvate kinase; PDHA-1, pyruvate dehydrogenase alpha subunit; PDHB-1, pyruvate dehydrogenase beta subunit; PCK-1/2, phosphoenolpyruvate carboxykinase; PYC-1, pyruvate carboxylase; MEN-1, malic enzyme; LDH-1, lactate dehydrogenase; GDH-1, glutamate dehydrogenase; GOT-2.2, glutamate oxoacetate transaminase.

**Fig. 8.**
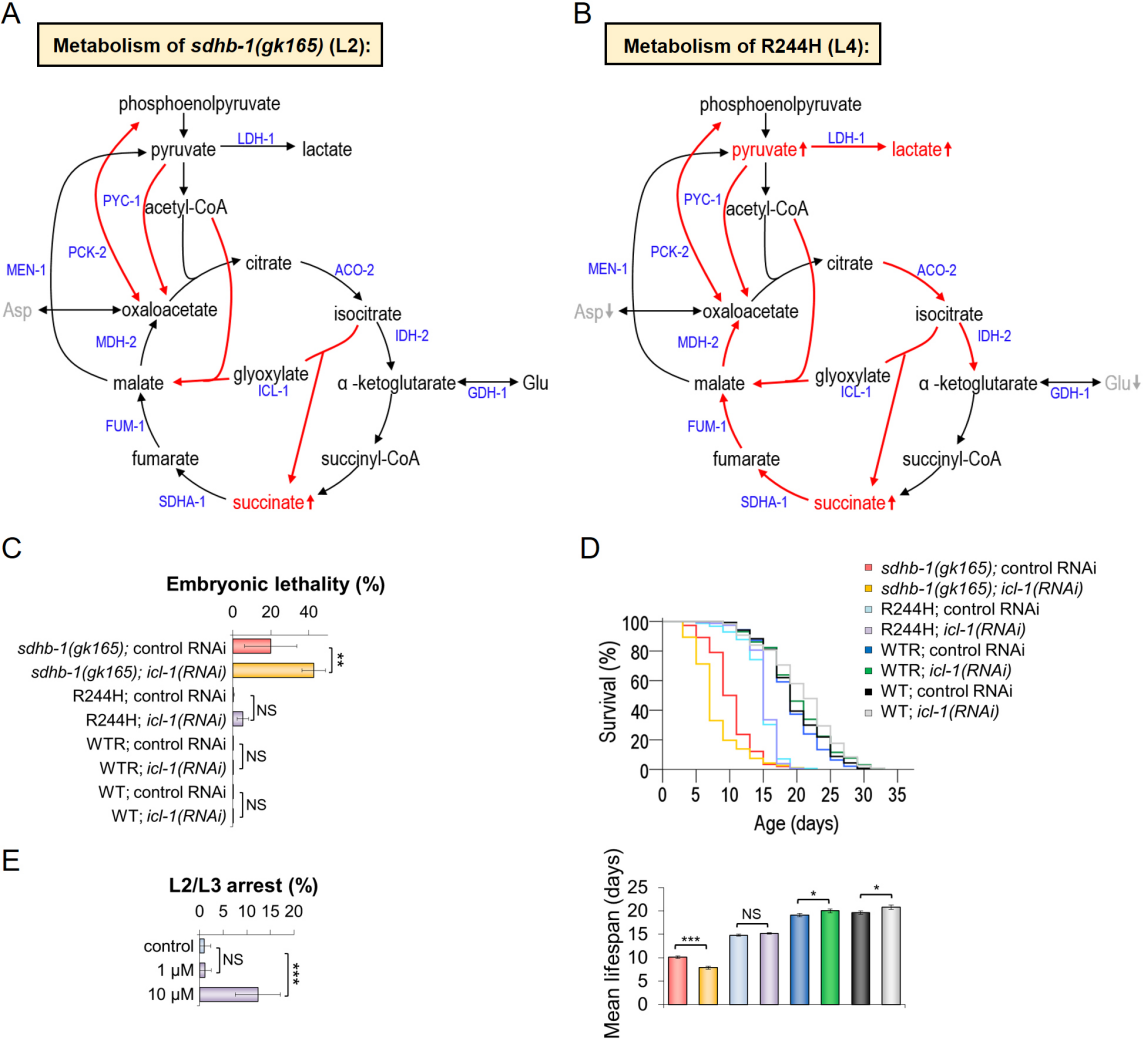
**Arg244His point mutants exhibit a rewired metabolism compared to *sdhb-1(gk165)* null mutants.** *icl-1(RNAi)* and LDH-1 inhibitor further enhance the premature developmental arrest of *gk165* deletional and R244H point mutants, respectively. (A,B) A model representing metabolic changes in *sdhb-1(gk165)* null mutants (A) and Arg244His point mutants (B). Both mutants display elevated glyoxylate cycle activity. Only point mutants partially use their TCA cycle while exhibiting high glycolytic activity. Expression of the genes encoding enzymes labeled in blue was analyzed by qPCR. Red arrows indicate high expression; black arrows indicate normal expression. (C,D) *icl-1(RNAi)* treatment affected *gk165* null mutants selectively: (C) embryonic lethality occurred in 42.5% of treated *gk165* homozygotes (*n*=353), whereas *gk165* animals fed HT115 bacteria expressing empty vector pPD129.36 (control RNAi) showed embryonic lethality in only 19.9% of the cases (*n*=315). (D) Lifespan of null mutant (*n*=188) and point mutant (*n*=155) animals was measured after ingestion of dsRNA specific for *icl-1*. Only null *sdhb-1(gk165)* animals showed a significantly shorter survival compared to their respective controls *sdhb-1(gk165)*;control RNAi (*n*=148) and R244H;control RNAi (*n*=155) (*n*=3 separate experiments). (E) R244H mutants were treated by LDH-A inhibitor GSK2837808A at 1 µM and 10 µM concentrations. The compound had no effect at the lower concentration, but at 10 µM 12.4% of treated animals demonstrated increased L2/L3 larval arrest. Statistical significance is indicated in each graph as ***P≤0.001, **P≤0.01 and *P≤0.05 versus the corresponding sample (NS, not significant). Results of embryonic lethality and LDH-A inhibitor treatment were analyzed by one-way ANOVA. Lifespan measurement was analyzed by independent two-sample Student's t-test with Bonferroni correction. Error bars represent s.e.

Next, we focused on genes related to the observed differences in the metabolic profiles of *sdhb-1* deletional and point mutants (Fig. 7A-C). Because our LC-MS data revealed a differentially compromised TCA cycle in *sdhb-1* null compared to point mutants (e.g. elevated succinate-to-fumarate ratio in both mutant backgrounds but a selective accumulation of pyruvate and lactate in point mutants), we first selected genes encoding TCA cycle-related enzymes and enzymes regulating lactate and pyruvate levels (Fig. 8A,B labeled in blue). First, we compared their expression levels in the null *sdhb-1(gk165)* and R244H mutants versus their stage-specific control strains (Fig. 7A,B). We observed an overlapping gene expression profile between the null and point mutants compared to their respective stage-matched controls. Specifically, we identified differences in the expression of aconitase (*aco-2*), isocitrate dehydrogenase (*idh-2*), isocitrate lyase/malate synthase of the glyoxylate cycle (*icl-1*), fumarase (*fum-1*), pyruvate carboxylase (*pyc-1*), phosphoenolpyruvate kinase (*pck-1/2*) and lactate dehydrogenase (*ldh-1*) compared to the appropriate stage-matched controls. In some instances, both mutants showed increased *icl-1*, *aco-2* and *pyc-1 pck-1/2* expression, whereas only null mutants showed decreased *fum-1* expression. The *fum-1* gene product controls the immediate fate of succinate as it is generated in the TCA cycle. Conversely, only the point mutants displayed elevated *ldh-1* levels compared to stage-matched L4 controls. This is consistent with our metabolic data (Fig. 7A,B) showing selective lactate accumulation in the point mutant.

Next, we used real-time quantitative PCR (qPCR) to validate expression levels of the above mentioned genes using *cdc-42* as a reference gene (Hoogewijs et al., 2008): expression levels were normalized to this gene using the ddCt method (Pfaffl, 2004). Genes were grouped by low, moderate and high expression relative to our transcriptomic analysis. In deletional mutants, the relatively lowly expressed genes included *sdha-1* and *fum-1*; the moderately expressed genes included glutamate dehydrogenase (*gdh-1*) and malic enzyme (*men-1*). In point mutants, highly selectively elevated expressed genes included *idh-2* and *ldh-1*, compared to *aco-2*, *icl-1*, *pyc-1* and *pck-1/2*, which were increased in both mutants (Fig. 7A,B; Table S8).

qPCR measurements largely confirmed previous transcriptomic data. Expression of *icl-1*, the bifunctional key enzyme of the glyoxylate shunt, showed the most remarkable change in both deletional and point mutants compared to the appropriate controls (∼20-fold or 10-fold change in deletional and point mutants, respectively; Fig. 7C; Table S9). qPCR data also confirmed results found in metabolic analysis: *ldh-1*, which generates lactate from pyruvate, was strongly and selectively increased in point mutant animals (Fig. 8A,B; Table S9). Interestingly, only R244H mutants showed increased *aco-2*, *idh-2*, *sdha-1*, *fum-1* and *mdh-2* expression. Both mutants showed elevated *pck-2* and *pyc-1* expression, which code for key enzymes of gluconeogenesis (Fig. 8A,B; Table S9).

### Effects of *icl-1(RNAi)* and a lactate dehydrogenase inhibitor on *sdhb-1* mutants

Next, we undertook some pilot experiments to determine whether our model is drug responsive. Based on our metabolomic data, the glyoxylate cycle plays an important role in the metabolism of *sdhb-1* mutants as we found that isocitrate lyase (*icl-1*) expression was significantly elevated in both mutant backgrounds (Fig. 7 and Fig. 8A,B). We first attempted to attenuate the glyoxylate shunt by silencing ICL-1 activity by feeding *sdhb-1* mutants with HT115 bacteria expressing double-stranded RNA (dsRNA) specific for *icl-1* [*icl-1(RNAi)*]*.* This influenced the lifespan and viability of *sdhb-1(-)* null mutants as follows: *icl-1(RNAi)* treatment resulted in partially penetrant lethality of *gk165* mutant embryos (Fig. 8C; Table S10) such that 42.5% of *gk165* animals treated with *icl-1(RNAi)* died as embryos, about double the controls (19.9%, fed with bacteria expressing the empty vector). Furthermore, *gk165* null mutants exposed to dsRNA specific for *icl-1* had significantly shorter lives compared to controls (Fig. 8D; Table S1). Interestingly, neither the lifespan nor the viability of R244H point mutants was affected by *icl-1(RNAi)* (Fig. 8C,D).

As point mutants showed a significant increase in lactate dehydrogenase (*ldh-1*) expression, we applied an inhibitor that targets the human LDH-1 homolog lactate dehydrogenase A (GSK2837808A) and is known to inhibit tumorigenesis in mouse models (Xie et al., 2014). GSK2837808A arrested the development of R244H point mutants at an earlier time point, such that R244H mutant embryos grown on bacteria and the LDH-A inhibitor at 10 µM concentration arrested development at the L2/L3 larval stage in 12.4% of cases, whereas the untreated controls reached adulthood but remained sterile (Fig. 8E; Table S11).

## DISCUSSION

In this work, our first aim was to create a tractable *in vivo* model of the SDH/complex II in the mitochondrial respiratory chain that catalyzes the oxidation of succinate to fumarate *pari passu* with the reduction of ubiquinone to ubiquinol. SDH is difficult to study because it has four subunits, encoded by separate genes (*SDHA-D*) that work in concert to link the TCA cycle to electron transport.

Our second aim was to determine whether a worm model would enable a better understanding of the different biological backgrounds following different *sdhb* mutations, only some of which are associated with an increased risk of malignant PPGL. Such a model may be of importance because current PPGL treatment options are limited. To that end, using bioinformatic tools, we also explored the structural consequences of one SDHB missense mutation that might affect the SDHA-SDHB interface.

Amongst SDHB mutations (Burnichon et al., 2009), we focused on worms bearing the Arg230His missense mutation that is causal of a familial form of PPGL. We generated two transgenic lines carrying the Arg244His missense mutation (corresponding to human Arg230His) and genomic wild-type *sdhb-1*. These transgenic lines were crossed into the *sdhb-1(gk165)* deletional background. As expected, only the wild-type *SDHB* transgene efficiently rescued the L2 larval arrest that is characteristic of the SDHB null mutant. In contrast, the point mutant corresponding to human Arg230His was unable to fully complement the deletional phenotype, producing only sterile adults with a significantly shortened lifespan of a similar degree to that of null mutants. These data are consistent with known lifespan-shortening effects of mutations in the *C. elegans* SDHC *mev-1* subunit (Chang et al., 2017).

To gain more insight into the nature of *gk165* and *Arg244His* mutant alleles, we examined whether SDHB is transcribed and translated in these mutant backgrounds. Wes was used to determine SDHB-1 status by applying a human anti-SDHB antibody, which successfully cross-reacted with the homologous worm protein. We first confirmed that *gk165* represents a true null allele as in this background SDHB-1 protein is absent. In *Arg244His* mutants, SDHB-1 is transcribed and translated, but at a lower level compared to the wild-type control. This result shows that the SDHB-1 R244H protein is stable enough to be detected, although it is functionally defective in the TCA cycle, given the elevated succinate-to-furmarate ratio. However, we do not know whether the SDH complex stays intact in the mutant or whether soluble SDHA is released with an attenuated function described as ‘CII low activity’ (Bezawork-Geleta et al., 2018). The status of SDHA needs to be examined in both *sdhb-1* deletional and point mutants in future studies.

In our metabolic studies, the *gk165* null allele showed the expected elevation of the SDH substrate succinate accompanied by a low fumarate level. Interestingly, point mutants showed an almost identically elevated succinate-to-fumarate ratio compared to deletional mutants, suggesting that point mutation also leads to loss of SDH activity in the TCA cycle. However, the mechanisms may be very different. Transcriptionally, only the null mutant displayed a reduction in both the SDHA and fumarase mRNA levels. Clearly, in the null state, elevated succinate cannot be further metabolized in the TCA cycle, but we saw no corresponding reduction in SDHA mRNA in the point mutant worm. Stimulated oxygen consumption was also equivalently reduced in both deletional and point mutant animals and was accompanied by downregulated mitochondrial and total ATP content, consistent with the notion that this related manifestation of SDH activity was eliminated equally in both mutants. These results are in line with published human data (Burnichon et al., 2009) and our bioinformatic predictions suggesting that the Arg244His substitution induces a conformational change in the structure of SDHB-1 that might result in either an inactive or altered enzyme. Although it is recognized that proteins are fairly robust and point mutations are unlikely to alter their 3D structure and function (Bowie et al., 1990), there are accumulating reports of several systems in which mutation of distal residues can lead to dramatic changes at a remote active site (Lee and Goodey, 2011), which often leads to aberrant and even inactive enzymes. Importantly, the guanidino group of Arg230 (Arg244 in worms) normally forms a conserved Asp224-Arg230 salt bridge that might be critical for the stability of SDHB proteins. The importance of this salt bridge is also supported by the fact that not only the Arg230His mutation described in this work, but also missense mutation of the bridging partner's histidine Asp224His, is linked to hereditary PPGL (Lefebvre et al., 2012).

Despite the above similarities in bioenergetics, the null and point mutants show significant developmental differences: the null worms show arrested development at the L2 larval stage, whereas point mutants develop further, eventually reaching adulthood, although they are then sterile. In order to understand this discrepancy, we carried out a relative metabolic and transcriptomic analysis of the two mutants.

We found elevated isocitrate lyase (*icl-1*) expression in both mutants (confirmed by qPCR, below). Isocitrate lyase is the key enzyme of the glyoxylate shunt, which cleaves the TCA intermediate isocitrate to generate the two products, glyoxylate and succinate. In the glyoxylate shunt, malate synthase then converts glyoxylate and acetyl-CoA into malate (Fig. 8A,B). The glyoxylate shunt is a variation of the TCA cycle, which centers on the conversion of acetyl-CoA to succinate, thereby enabling the synthesis of carbohydrates. This action transects the TCA by bypassing the steps that normally liberate carbon dioxide as aKG catabolized to succinate. The glyoxylate shunt functions in plants, some microorganisms and nematodes, but it remains controversial as to whether it can function in humans (Strittmatter et al., 2014). In wild-type worms, the glyoxylate cycle is active during early development (at the pre-hatching embryonic development and at the first L1 larval stages), peaks at L2, and then declines thereafter in L3-L4 larvae through to adulthood (Liu et al., 1997). Energy production through the TCA cycle shows an opposite tendency as its activity is elevated after hatching and increases substantially throughout L1-L4 larval development (Wadsworth and Riddle, 1989). The metabolic switch between the (early) glyoxylate shunt and the (later) TCA cycle pivots at the L2/L3 larval stage, exactly when we observed arrested development in *sdhb-1(gk165)* deletional mutants.

Correspondingly, transcriptomic and qPCR data showed elevated *icl-1* expression in deletional mutants, which arrest at L2 larval stage, in which the glyoxylate cycle is active. *icl-1* expression was also increased in Arg244His point mutants, which might compensate for the absence of SDH activity in the TCA cycle. Interestingly, key enzymes of gluconeogenesis (*pck-2*, *pyc-1*) were not significantly different when point mutants were compared to the null state. Neither was the relative expression of the message for several TCA cycle enzymes (*ogdh-1*, *suca-1*, *fum-1* and *mdh-2*) different in R244H worms compared to wild-type controls, which suggests that the point mutants can express key components of the TCA cycle in addition to the glyoxylate cycle.

Conversely, gene expression studies showed important differences in the metabolism of null versus point mutant worms. Transcriptomics also demonstrated significant metabolic alterations, consistent with LC-MS measurements in the point and deletional mutants. Increased expression of the message for the enzyme interconverting lactate and pyruvate (*ldh-1*) was confined to point mutants, consistent with our biochemical data, which showed high lactate levels only in the Arg244His point mutants. Together, the data suggest that Arg244His point mutants, which develop into sterile adults, exhibit a higher glycolytic activity alongside a partially functioning TCA cycle, where the glyoxylate shunt likely compensates for the absence of SDH activity. However, null mutants (which show arrested development at the L2 larval stage) have no similarly rewired metabolism and can only use the glyoxylate cycle, which might explain their early arrested development. In this respect, in Arg244His mutants, the glycolytic-oxphos metabolite concentrations/ratios were reminiscent of tumor cells undergoing Warburg effect, i.e. favoring glycolysis even under aerobic conditions instead of oxidative phosphorylation (Vander Heiden et al., 2009). Consistent with this notion, the Warburg effect was shown to be induced in human hepatocellular carcinoma cells in response to decreased succinate dehydrogenase B activity (Tseng et al., 2018).

In the work reported here, we have generated a novel nematode model for examining and evaluating the developmental and metabolic consequences of two clinically important *SDHB* mutations (null and point mutant). We observed very different metabolic alterations as a result of *sdhb-1* deletion in comparison to an equally cancer-proven but highly malignant (Arg230His) missense mutation.

We propose that such a worm model could, in principle, be used to test the effect of different drug candidates to see whether they act selectively on mutant worms, leaving both normal and null worms unscathed. In a pilot experiment, we targeted the glyoxylate shunt and lactate metabolism in our model. In order to attenuate the glyoxylate shunt, we inactivated, by RNAi, *icl-1*, which encodes isocitrate lyase, the key enzyme of the shunt. We found that *icl-1(RNAi)* treatment selectively acted on *gk165* null mutants, caused a partially penetrant embryonic lethality and shortened lifespan. These data are consistent with the idea that the glyoxylate cycle serves a critical role in null mutants by sustaining their development until the L2 stage. However, R244H point mutants, which are able to reach adulthood, specifically display an elevated lactate dehydrogenase (*ldh-1*) expression in addition to the increased *icl-1* message. We tested the notion that these point mutated animals use lactate as an energy source as a consequence of an increased LDH-1 production, by pharmacologically inhibiting LDH-1 activity using an established LDH-A inhibitor previously studied in human cells. LDH-1 inhibition resulted in an earlier than expected L2/L3 larval arrest of point mutants, showing that LDH-1 activity is indeed crucial in their development.

Thus, we have shown that our novel PPGL model is potentially druggable. Our modeling approach might also be of benefit to research groups studying other SDH mutations and different TCA cycle genes linked to metabolic diseases and cancer.

During the preparation of this paper, Lotti et al. (2019) extended the tripartite classification of PPGL (see Introduction) using a developmentally based approach to PPGL ontogeny. They examined the morphology of PPGL and propose that ‘paraganglioma is a fundamentally organized, albeit aberrant, tissue composed of neoplastic vascular and neural cell types that share a common origin from a multipotent mesenchymal-like stem/progenitor cell’ (Lotti et al., 2019). Our developmental paradigm provides a tractable model to test this idea and the classical classification system using tractable (and potentially druggable) *C. elegans.*

## MATERIALS AND METHODS

### *C. elegans* strains

*C. elegans* strains were maintained under standard conditions (Brenner, 1974). The following strains were used: wild type (WT), Bristol N2; VC294, {*sdhb-1(gk165)/ mIn1 (mIs14 [myo-2::gfp; pes-10::gfp]) dpy-10(e128)] II.*}; COP952, [*pNU636 (Psdhb-1_sdhb-1(genomic G731A)_UTRsdhb-1; unc-119(+)) X.; unc-119(ed3) III.*]; COP957, [*pNU637 (Psdhb-1_sdhb-1(genomic wt)_UTRsdhb-1; unc-119(+)) X.; unc-119(ed3) III.*]; R244H, *sdhb-1(gk165)/ mIn1 [mIs14 [myo-2::gfp; pes-10::gfp] dpy-10(e128)] II.; unc-119(ed3) III.(?); pNU636 (Psdhb-1_sdhb-1(G731A)_UTRsdhb-1; unc-119(+)) X.*; WTR, *sdhb-1(gk165)/ sdhb-1(gk165) II.; unc-119(ed3) III.(?); pNU637 (Psdhb-1_sdhb-1(genomic wt)_UTRsdhb-1; unc-119(+)) X*.; *sdhb-1p::gfp* reporter strain, *unc-119(ed3)III; Ex[psdhb-1::gfp + unc-119(+)]*.

### Genotyping of mutant strains

For genotyping the *gk165* deletion, we used the following forward and reverse primers: 5′-TGAAATTGCATTGTCCTCTCAC-3′ and 5′-CGATTTGATCTCTCCGATGG-3′ (Table S12). In this study, we also used two transgenic strains generated by Knudra Transgene Service: *sdhb-1* wild-type transgenic strain [*sdhb-1p::SDHB-1(wt)::sdhb-1u*] and a transgenic strain carrying the Arg244His missense mutation (caused by a G731A point mutation in the coding sequence) [*sdhb-1p::SDHB-1(G731A)::sdhb-1u*]. Both strains carry the corresponding single-copy transgene inserted into a given region of the X chromosome by MosSCI. For genotyping the transgenes, we used the following primers: F1, 5′-GATCAAATCGCTGCTTACTG-3′; F2, 5′-CATTTGATCAGCGTAGTTGC-3′; R1, 5′-ATTCTTTCTCCTAGCCTTCG-3′ (Table S12). F1+R1 amplifies a 770 bp transgene-specific product (the product from the wild type is too large to be amplified), while amplification by F2+R1 gives a 460 bp wild-type-specific product.

### Lifespan assay

Lifespan assays were conducted at 20°C on nematode growth medium (NGM) plates seeded with *Escherichia* *coli* [OP50 or HT115(DE3)] bacteria. The worms were synchronized by NaOH/hypochlorite treatment. Three to four parallel plates with 40 animals of the corresponding strains were used. The numbers of animals used for the experiment are shown (Table S1). Animals that climbed up the wall of plastic dishes or died prematurely because of picking were excluded/censored from the analysis. Animals were scored as dead when they no longer moved when pushed a few times. IBM SPSS Statistics software was used to carry out statistical analysis. The *P*-values for comparing Kaplan–Meyer survival curves between two groups were determined using log-rank tests. The *P*-values for comparing mean lifespans were determined using independent two-sample Student's *t*-tests with Bonferroni correction.

### Wes

Protein lysates of worms of different genotypes were prepared as described in Farkas et al. (2019). The protein content of worm lysates was quantified by Bradford protein assay (Bio-Rad) and immunodetected with Wes Simple analysis on Wes system (ProteinSimple-Biotechne 004-600). Then, 12-230 kDa Separation Module (ProteinSimple SM-W004) and an Anti-Rabbit Detection Module (ProteinSimple DM-001) were applied. Briefly, protein samples were diluted in sample buffer thereafter mixed with Fluorescent Master Mix 1:4 and denatured at 95°C for 5 min. According to the manufacturer's instructions, the samples, blocking reagent (antibody diluent), primary antibodies, horseradish peroxidase (HRP)-conjugated secondary antibodies and chemiluminescent substrate were added to the plate. Immunodetection was performed automatically, and the results are reported as virtual gels based on chemiluminescence signals. The default settings of the device were the following: stacking and separation at 375 V for 25 min; blocking reagent for 30 min; primary and secondary antibodies both for 30 min; luminol/peroxide chemiluminescence detection for 30 min (exposure times were selected for the antibodies between 1 s and 512 s). The following primary antibodies were used: rabbit polyclonal anti-DYN-1 (1:100; kindly provided by Zheng Zhou, Baylor College of Medicine, Houston, TX, USA, rabbit anti-SDHB (1:50; GTX104628, Genetex).

### Generation of a transgenic strain

The *sdhb-1* minimal promoter fragment was amplified using primers listed in Table S12 and was digested with AscI and NotI, then cloned into pRH21 (Hofmann et al., 2002). Subsequently, transgenic worms were generated by biolistic transformation using the *unc-119(ed3)* mutant genetic background and the *unc-119(+)* gene as a co-transformation marker.

### LC-MS

Worms at different larval stages were picked to Eppendorf tubes containing M9 solution (in order to prevent any effect of *E. coli* metabolites on the measurements). Samples were centrifuged at 15,294 ***g*** at 4°C for 3 min, after which the supernatant was discarded and the pellet was placed to liquid nitrogen until it became frozen. Metabolite extraction was performed by adding 450 μl methanol:chloroform:water (9:1:1 v/v) solution to the pellets, which were then homogenized and vortexed for a minute. Next, the pellet was centrifuged at 20,817 ***g*** at 4°C for 10 min. The supernatant was transferred to a new Eppendorf tube and was stored at −80°C until measurement was performed. LC-MS/MS assays were conducted using a Perkin-Elmer Flexar FX10 ultra-performance liquid chromatograph coupled with a Sciex 5500 QTRAP mass spectrometer. For chromatographic separation, a Phenomenex Luna Omega C18 column (100×2.1 mm, 1.6 µm) was used (Gen-Lab Ltd, Budapest, Hungary). The mobile phase consisted of water containing 0.1% (v/v) formic acid (A) and methanol containing 0.1% (v/v) formic acid (B). The following gradient program was used: 100% A, hold 0.2 min, ramp A to 10% in 9.8 min, immediately ramp A back to 100% in 1.0 min, hold 7.0 min. The total run time was 18.0 min, the flow rate was 180 µl/min and 5 µl sample was injected. Column temperature was set at 35°C. The mass spectrometer was set to negative electrospray ionization mode using the following parameters: source temperature, 300°C; ionization voltage, −4000 V; curtain gas, 35 psi; gas1, 35 psi; gas2, 35 psi; entrance potential, −10 V; CAD gas, medium.

Quantitative analysis was performed in multiple reaction monitoring (MRM) mode. The following metabolites were detected: citrate, fumarate, lactate, malate, pyruvate, succinate, aspartate, glutamate. IBM SPSS Statistics software was used to carry out statistical analysis and determine means±s.d. using one-way ANOVA.

### Oxygen consumption measurement by Seahorse XF Analyzer

The Seahorse XF96 Analyzer (Agilent, USA) was used for assessment of worm bioenergetics. Hydration of probes was performed with 200 µl Seahorse Bioscience XF96 calibrant (pH 7.4) solution at 37°C overnight. Heat and temperature control on the Seahorse XF96 Analyzer was switched off and the experiment was carried out in a cold room of 10°C in order to ensure a temperature of 20°C inside the analyzer. Worms were picked into M9-filled 96-well Seahorse plates (L4 larval stage, 20 worms per well; L2 larval stage, 100 worms per well). We used two compounds, FCCP at 10 µM final well concentration and sodium azide at 40 mM final well concentration, to assess bioenergetics in *sdhb-1* mutant worms and control strains. FCCP is a protonophore and uncoupler reagent of mitochondrial oxidative phosphorylation, which uncouples ATP synthesis (phosphorylation) from the electron transport chain (oxidation) by transporting H^+^ ions through the mitochondrial membrane bypassing the ATP synthase. Sodium azide is a complex IV (cytochrome *c* oxidase) inhibitor, which binds directly to the heme prosthetic group of cytochrome *c* oxidase to prevent the final transfer of electrons to oxygen. It also inhibits the reverse mode of ATP synthase. First, the total cellular OCR was measured, from which basal respiration can be derived by subtracting non-mitochondrial oxygene consumption. In our experiments, 100 μM FCCP was injected to obtain maximal respiration, then 400 mM sodium azide was used to allow measurements of the non-mitochondrial respiration. Spare capacity was calculated based on the difference between the basal respiration and maximal respiration. IBM SPSS Statistics software was used to carry out statistical analysis and determine means±s.d. using one-way ANOVA.

### Mitotracker

A synchronized population of worms was obtained after embryo isolation, then washed three times with M9 buffer. The worms were incubated with the Mitotracker dye (Mitotracker Red CMX Ros, Sigma-Aldrich) for 30 min at 22°C, then washed three times with M9 buffer. Randomly selected worms were observed under confocal microscopy at 40× after being paralyzed with 2% sodium azide solution. This technique measures the relative fold-change intensity of mitochondrial content. IBM SPSS Statistics software was used to carry out statistical analysis and determine means±s.d. using two-sample Student's *t*-tests.

### ATP assay

After obtaining a synchronized population of worms, 2000 worms from each group (obtained by normalized approximation procedure for worm number) were homogenized in a sonicator and the supernatant was collected. A Bradford assay was performed to estimate the protein content in the supernatant. For each group, 20 μg of the protein was collected, to which 200 μl of ATP assay mix was added. The luminescence was immediately read using a Perkin Elmer (Victor) luminometer. IBM SPSS Statistics software was used to carry out statistical analysis and determine means±s.d. using two-sample Student's *t*-tests.

### Modeling the human SDHA/B complex

The multiple alignment was generated using P21912|SDHB_HUMAN Succinate dehydrogenase B as query to perform a blastp search using the BLAST tools of NCBI. Top-matching sequences, annotated as succinate dehydrogenase B, were selected and were aligned with the Constraint-based Multiple Alignment Tool of NCBI (Fig. 6A). Subunits A and B of porcine SDH (PDB ID: 4YTP) were used to model human SDHA/B complex (97% sequence identity to human SDHA/B; see Fig. S5 for alignment). Transit sequences were omitted in the modeling process. The wild-type and R230H variants of human SDHA/B complex were constructed using homology modeling. Modeller (Sali and Blundell, 1993) was employed to generate 1000 homology models for each of the wild-type and R230H mutant. Twenty models were selected from the 1000 models: the top ten models selected by the DOPE scoring function (Shen and Sali, 2006) and the top ten models selected by Modeller's probability density function. Inter-residue pairwise contacts were calculated using an in-house Python implementation. Two residues were deemed to be in contact if any two heavy atoms from the two residues were less than 4.5 Å apart. Averaged contact frequency was evaluated by averaging the corresponding residue-pair contacts over the 20 models. Differences in averaged residue-pair contact frequencies were used to quantify the structural changes upon mutation.

### Transcriptomics analysis

#### RNA sequencing (RNA-seq)

The total nucleic acid extraction of wild-type and mutant animals was conducted by TRI Reagent^®^ (Molecular Research Center, Cincinnati, OH, USA) as recommended by the manufacturer. The crude extract was further purified with RNeasy Mini Kit (Qiagen, Hilden, Germany) using the manufacturer's protocol. To remove DNA contamination that might interfere with downstream applications, Column DNase treatment with Qiagen RNase-free DNase kit was applied. To purify pure mature polyA-tailed mRNA, we performed a magnetic bead-based separation on the purified total RNA with NEB NEXT Poly(A) mRNA magnetic Isolation Module (New England Biolabs, Ipswich, MA, USA). We used 100 ng mature polyA-tailed mRNA per sample to construct RNA-seq libraries. The NEB NEXT ULTRA II Directional RNA Library Preparation Kit (Illumina, San Diego, CA, USA) was used to create sample libraries using the NEB factory protocol. Each sample was individually indexed with NEB NEXT Multiplex Oligos for the Illumina Kit. Samples were equimolarly pooled to a 4 nM sequencing library and sequenced with Illumina MiSEQ v2 500 cycles reagent on the Illumina MiSEQ System.

The *C. elegans* WBcel235 genome release 94 assemblies were obtained from the Ensembl database. The chromosomal sequences and annotation files were downloaded as FASTA and GTF format, respectively. Genome and transcriptome sequences in FASTA format were indexed with Tophat2 (Bowtie2). Illumina paired-end sequencing data were exported in FASTQ file format.

The FASTQC program (https://www.bioinformatics.babraham.ac.uk/projects/fastqc/) was used to pre-process RNA-seq data, to evaluate the qualities of original and trimmed reads (Martin, 2011). The trimmed sequence sets were aligned to the genome with Tophat2 (Bowtie2), using default parameters (Langmead et al., 2009; Kim et al., 2013). For further analysis, the reads were sorted by Samtools (http://samtools.sourceforge.net) according to coordinates. The function feature counts (Rsubread R package) were used for counting reads to genomic features. Two variants of count-based gene expression level estimation union-exon and transcript-based counting were used. To implement union-exon, we selected the exon entries from the annotation files, grouped them by the Ensembl gene identifier, and merged overlapping exons for each gene using the function to summarize overlaps from bioconductor library GenomicAlignments. We then generated per-gene counts by intersecting the genomic alignments with the resulting ‘pseudoexons’ using featureCount (Liao et al., 2014).

The Bioconductor package edgeR was used for differential expression analyses of read counts for genomic features arising from RNA-seq (Robinson et al., 2010; McCarthy et al., 2012). IBM SPSS Statistics software was used to carry out statistical analysis and determine means±s.d. using two-sample Student's *t*-tests with Bonferroni correction.

#### Transcriptomics validation

For validation of the gene expression data calculated in the transcriptomic analysis a reverse transcription (RT)-qPCR approach was used. Primers were designed for candidate genes [*cdc-42* (reference gene), *aco-2*, *fum-1*, *gdh-1*, *icl-1*, *idh-2*, *ldh-1*, *men-1*, *pck-2*, *pyc-1*, *sdha-1*] on exon boundaries suitable for SYBR GREEN assay using Primer3 (Table S12). Primers were manufactured by Eurofins Genomics AT GmbH. RNA was prepared as described above and 100 ng was transcribed to cDNA with a High-Capacity RNA-to-cDNA Kit (Thermo Fisher Scientific, Waltham, MA, USA). cDNA was assayed with a SensiFAST SYBR Lo-ROX Kit (Bioline, London, UK) and using specific primers spanning exon boundaries of respective genes on a Quant Studio 7 FLEX Q-RT-PCR system (Thermo Fisher Scientific). Relative gene expression was estimated by the ddCt method (Pfaffl, 2004). IBM SPSS Statistics software was used to carry out statistical analysis and determine means±s.d. using one-way ANOVA.

### RNAi

The *icl-1(gei-7)* PCR fragment was amplified using primers listed in Table S12. Next, it was cloned into L4440 vector (pPD129.36; Fire Lab 1997 Vector Kit) by SacI and Acc65I. The obtained construct was transformed into bacterial strain HT115(DE3). RNAi experiments were performed at 20°C as described (Timmons, 2006).

### LDH-A inhibitor treatment

R244H animals were treated by LDH-A inhibitor GSK2837808A (Tocris) at 1 µM and 10 µM (Xie et al., 2014). R244H strain was synchronized by NaOH/hypochlorite treatment, and embryos were put on NGM plates with a seeded lawn of OP50 bacteria containing GSK2837808A at a final concentration of 1 µM and 10 µM.

## Consent to participate

For the family tree mentioned in this article, we contacted members of the family through Findacure Foundation and the Phaeo Para Cancer Charity. They provided consent and their phenotypic data.

## Supplementary Material

Supplementary information

## References

[DMM044925C1] Amar L., Bertherat J., Baudin E., Ajzenberg C., Bressac-de Paillerets B., Chabre O., Chamontin B., Delemer B., Giraud S., Murat A. et al. (2005). Genetic testing in pheochromocytoma or functional paraganglioma. *J. Clin. Oncol.* 203, 8812-8818. 10.1200/JCO.2005.03.1484 16314641

[DMM044925C2] Benn D. E., Robinson B. G. and Clifton-Bligh R. J. (2015). 15 YEARS OF PARAGANGLIOMA: Clinical manifestations of paraganglioma syndromes types 1-5. *Endocr Relat. Cancer* 22, T91-T103. 10.1530/ERC-15-0268 26273102 PMC4532956

[DMM044925C3] Bezawork-Geleta A., Wen H., Dong L., Yan B., Vider J., Boukalova S., Krobova L., Vanova K., Zobalova R., Sobol M. et al. (2018). Alternative assembly of respiratory complex II connects energy stress to metabolic checkpoints. *Nat. Commun.* 9, 2221 10.1038/s41467-018-04603-z 29880867 PMC5992162

[DMM044925C4] Bowie J. U., Reidhaar-Olson J. F., Lim W. A. and Sauer R. T. (1990). Deciphering the message in protein sequences: tolerance to amino acid substitutions. *Science* 247, 1306-1310. 10.1126/science.2315699 2315699

[DMM044925C5] Brenner S. (1974). The genetics of Caenorhabditis elegans. *Genetics* 77, 71-94. 4366476 10.1093/genetics/77.1.71 PMC1213120

[DMM044925C6] Brouwers F. M., Eisenhofer G., Tao J. J., Kant J. A., Adams K. T., Linehan W. M. and Pacak K. (2006). High frequency of SDHB germline mutations in patients with malignant catecholamine-producing paragangliomas: implications for genetic testing. *J. Clin. Endocrinol. Metab.* 91, 4505-4509. 10.1210/jc.2006-0423 16912137

[DMM044925C7] Burnichon N., Rohmer V., Amar L., Herman P., Leboulleux S., Darrouzet V., Niccoli P., Gaillard D., Chabrier G., Chabolle F. et al. (2009). The succinate dehydrogenase genetic testing in a large prospective series of patients with paragangliomas. *J. Clin. Endocrinol. Metab.* 94, 2817-2827. 10.1210/jc.2008-2504 19454582

[DMM044925C8] Cascón A., López-Jiménez E., Landa I., Leskelä S., Leandro-García L. J., Maliszewska A., Letón R., de la Vega L., García-Barcina M. J., Sanabria C. et al. (2009). Rationalization of genetic testing in patients with apparently sporadic pheochromocytoma/paraganglioma. *Horm. Metab. Res.* 41, 672-675. 10.1055/s-0029-1202814 19343621

[DMM044925C9] Cerecer-Gil N. Y., Figuera L. E., Llamas F. J., Lara M., Escamilla J. G., Ramos R., Estrada G., Hussain A. K., Gaal J., Korpershoek E. et al. (2010). Mutation of SDHB is a cause of hypoxia-related high-altitude paraganglioma. *Clin. Cancer Res.* 16, 4148-4154. 10.1158/1078-0432.CCR-10-0637 20592014

[DMM044925C10] Chang H.-W., Pisano S., Chaturbedi A., Chen J., Gordon S., Baruah A. and Lee S. S. (2017). Transcription factors CEP-1/p53 and CEH-23 collaborate with AAK-2/AMPK to modulate longevity in Caenorhabditis elegans. *Aging Cell* 16, 814-824. 10.1111/acel.12619 28560849 PMC5506430

[DMM044925C11] Farkas Z., Petric M., Liu X., Herit F., Rajnavolgyi E., Szondy Z., Budai Z., Orban T. I., Sandor S., Mehta A. et al. (2019). The nucleoside diphosphate kinase NDK-1/NME1 promotes phagocytosis in concert with DYN-1/Dynamin. *FASEB J.* 33, 11606-11614. 10.1096/fj.201900220R 31242766 PMC6819981

[DMM044925C12] Gunawardane P. T. K. and Grossman A. (2017). Phaeochromocytoma and Paraganglioma. *Adv. Exp. Med. Biol.* 956, 239-259. 10.1007/5584_2016_76 27888488

[DMM044925C13] Guzy R. D., Sharma B., Bell E., Chandel N. S. and Schumacker P. T. (2008). Loss of the SdhB, but Not the SdhA, subunit of complex II triggers reactive oxygen species-dependent hypoxia-inducible factor activation and tumorigenesis. *Mol. Cell. Biol.* 28, 718-731. 10.1128/MCB.01338-07 17967865 PMC2223429

[DMM044925C14] Hes F. J., Weiss M. M., Woortman S. A., de Miranda N. F., van Bunderen P. A., Bonsing B. A., Stokkel M. P., Morreau H., Romijn J. A., Jansen J. C. et al. (2010). Low penetrance of a SDHB mutation in a large Dutch paraganglioma family. *BMC Med. Genet.* 11, 92 10.1186/1471-2350-11-92 20540712 PMC2891715

[DMM044925C15] Hofmann E. R., Milstein S., Boulton S. J., Ye M., Hofmann J. J., Stergiou L., Gartner A., Vidal M. and Hengartner M. O. (2002). Caenorhabditis elegans HUS-1 is a DNA damage checkpoint protein required for genome stability and EGL-1-mediated apoptosis. *Curr. Biol.* 12, 1908-1918. 10.1016/S0960-9822(02)01262-9 12445383

[DMM044925C16] Hoogewijs D., Houthoofd K., Matthijssens F., Vandesompele J. and Vanfleteren J. R. (2008). Selection and validation of a set of reliable reference genes for quantitative sod gene expression analysis in C. elegans. *BMC Mol. Biol.* 9, 291 10.1186/1471-2199-9-9 PMC2254638 18211699

[DMM044925C17] Huang J. and Lemire B. D. (2009). Mutations in the C. elegans succinate dehydrogenase iron-sulfur subunit promote superoxide generation and premature aging. *J. Mol. Biol.* 387, 559-569. 10.1016/j.jmb.2009.02.028 19233206

[DMM044925C18] Hujber Z., Horváth G., Petővári G., Krencz I., Dankó T., Mészáros K., Rajnai H., Szoboszlai N., Leenders W. P. J., Jeney A. et al. (2018). GABA, glutamine, glutamate oxidation and succinic semialdehyde dehydrogenase expression in human gliomas. *J. Exp. Clin. Cancer Res.* 37, 271 10.1186/s13046-018-0946-5 30404651 PMC6223071

[DMM044925C19] Inaoka D. K., Shiba T., Sato D., Balogun E. O., Sasaki T., Nagahama M., Oda M., Matsuoka S., Ohmori J., Honma T. et al. (2015). Structural Insights into the Molecular Design of Flutolanil Derivatives Targeted for Fumarate Respiration of Parasite Mitochondria. *Int. J. Mol. Sci.* 16, 15287-15308. 10.3390/ijms160715287 26198225 PMC4519900

[DMM044925C20] Kantorovich V. and Pacak K. (2018). New insights on the pathogenesis of paraganglioma and pheochromocytoma. *F1000Res* 7 10.12688/f1000research.14568.1 PMC6173107 30345003

[DMM044925C21] Kim D., Pertea G., Trapnell C., Pimentel H., Kelley R. and Salzberg S. L. (2013). TopHat2: accurate alignment of transcriptomes in the presence of insertions, deletions and gene fusions. *Genome Biol.* 14, R36 10.1186/gb-2013-14-4-r36 23618408 PMC4053844

[DMM044925C22] Koopman M., Michels H., Dancy B. M., Kamble R., Mouchiroud L., Auwerx J., Nollen E. A. and Houtkooper R. H. (2016). A screening-based platform for the assessment of cellular respiration in Caenorhabditis elegans. *Nat. Protoc.* 11, 1798-1816. 10.1038/nprot.2016.106 27583642 PMC5040492

[DMM044925C23] Langmead B., Trapnell C., Pop M. and Salzberg S. L. (2009). Ultrafast and memory-efficient alignment of short DNA sequences to the human genome. *Genome Biol.* 10, R25 10.1186/gb-2009-10-3-r25 19261174 PMC2690996

[DMM044925C24] Lee J. and Goodey N. M. (2011). Catalytic contributions from remote regions of enzyme structure. *Chem. Rev.* 111, 7595-7624. 10.1021/cr100042n 21923192

[DMM044925C25] Lefebvre S., Borson-Chazot F., Boutry-Kryza N., Wion N., Schillo F., Peix J. L., Brunaud L., Finat A., Calender A. and Giraud S. (2012). Screening of mutations in genes that predispose to hereditary paragangliomas and pheochromocytomas. *Horm. Metab. Res.* 44, 334-338. 10.1055/s-0032-1306308 22517554

[DMM044925C26] Liao Y., Smyth G. K. and Shi W. (2014). featureCounts: an efficient general purpose program for assigning sequence reads to genomic features. *Bioinformatics* 30, 923-930. 10.1093/bioinformatics/btt656 24227677

[DMM044925C27] Lin H., Su X. and He B. (2012). Protein lysine acylation and cysteine succination by intermediates of energy metabolism. *ACS Chem. Biol.* 7, 947-960. 10.1021/cb3001793 22571489 PMC3376250

[DMM044925C28] Liu F., Thatcher J. D. and Epstein H. F. (1997). Induction of glyoxylate cycle expression in Caenorhabditis elegans: a fasting response throughout larval development. *Biochemistry* 36, 255-260. 10.1021/bi9623800 8993341

[DMM044925C29] Lotti L. V., Vespa S., Pantalone M. R., Perconti S., Esposito D. L., Visone R., Veronese A., Paties C. T., Sanna M., Verginelli F. et al. (2019). A Developmental Perspective on Paragangliar Tumorigenesis. *Cancers* 11, 273 10.3390/cancers11030273 PMC6468609 30813557

[DMM044925C30] Luz A. L., Rooney J. P., Kubik L. L., Gonzalez C. P., Song D. H. and Meyer J. N. (2015). Mitochondrial Morphology and Fundamental Parameters of the Mitochondrial Respiratory Chain Are Altered in Caenorhabditis elegans Strains Deficient in Mitochondrial Dynamics and Homeostasis Processes. *PLoS One* 10, e0130940. 26106885 10.1371/journal.pone.0130940 PMC4480853

[DMM044925C31] Martin M. (2011). Cutadapt removes adapter sequences from high-throughput sequencing reads. 17, 3 10.14806/ej.17.1.200

[DMM044925C32] McCarthy D. J., Chen Y. and Smyth G. K. (2012). Differential expression analysis of multifactor RNA-Seq experiments with respect to biological variation. *Nucleic Acids Res.* 40, 4288-4297. 10.1093/nar/gks042 22287627 PMC3378882

[DMM044925C33] Murphy M. P. and O'Neill L. A. J. (2018). Krebs Cycle Reimagined: The Emerging Roles of Succinate and Itaconate as Signal Transducers. *Cell* 174, 780-784. 10.1016/j.cell.2018.07.030 30096309

[DMM044925C34] Pacak K. and Tella S. H. (2000). Pheochromocytoma and Paraganglioma. In *Endotext* (eds Feingold K. R., Anawalt B., Boyce A., Chrousos G., Dungan K., Grossman A., Hershman J. M., Kaltsas G., Koch C. and Kopp P. et al.). South Dartmouth, MA: MDText.com, Inc.

[DMM044925C35] Pfaffl M. W. (2004). Quantification strategies in real-time PCR. In *A–Z of Quantitative PCR* (ed. Bustin S.A.). International University Line (IUL): La Jolla, CA, USA.

[DMM044925C36] Pollard P. J., Briere J. J., Alam N. A., Barwell J., Barclay E., Wortham N. C., Hunt T., Mitchell M., Olpin S., Moat S. J. et al. (2005). Accumulation of Krebs cycle intermediates and over-expression of HIF1alpha in tumours which result from germline FH and SDH mutations. *Hum. Mol. Genet.* 14, 2231-2239. 10.1093/hmg/ddi227 15987702

[DMM044925C37] Pujol C., Bratic-Hench I., Sumakovic M., Hench J., Mourier A., Baumann L., Pavlenko V. and Trifunovic A. (2013). Succinate dehydrogenase upregulation destabilize complex I and limits the lifespan of gas-1 mutant. *PLoS One* 8, e59493 10.1371/journal.pone.0059493 23555681 PMC3610896

[DMM044925C38] Robinson M. D., McCarthy D. J. and Smyth G. K. (2010). edgeR: a Bioconductor package for differential expression analysis of digital gene expression data. *Bioinformatics* 26, 139-140. 10.1093/bioinformatics/btp616 19910308 PMC2796818

[DMM044925C39] Rustin P. and Rotig A. (2002). Inborn errors of complex II--unusual human mitochondrial diseases. *Biochim. Biophys. Acta.* 1553, 117-122. 10.1016/S0005-2728(01)00228-6 11803021

[DMM044925C40] Šali A. and Blundell T. L. (1993). Comparative protein modelling by satisfaction of spatial restraints. *J. Mol. Biol.* 234, 779-815. 10.1006/jmbi.1993.1626 8254673

[DMM044925C41] Selak M. A., Armour S. M., MacKenzie E. D., Boulahbel H., Watson D. G., Mansfield K. D., Pan Y., Simon M. C., Thompson C. B. and Gottlieb E. (2005). Succinate links TCA cycle dysfunction to oncogenesis by inhibiting HIF-alpha prolyl hydroxylase. *Cancer Cell* 7, 77-85. 10.1016/j.ccr.2004.11.022 15652751

[DMM044925C42] Senoo-Matsuda N., Yasuda K., Tsuda M., Ohkubo T., Yoshimura S., Nakazawa H., Hartman P. S. and Ishii N. (2001). A defect in the cytochrome b large subunit in complex II causes both superoxide anion overproduction and abnormal energy metabolism in Caenorhabditis elegans. *J. Biol. Chem.* 276, 41553-41558. 10.1074/jbc.M104718200 11527963

[DMM044925C43] Shen M. Y. and Sali A. (2006). Statistical potential for assessment and prediction of protein structures. *Protein Sci.* 15, 2507-2524. 10.1110/ps.062416606 17075131 PMC2242414

[DMM044925C44] Slane B. G., Aykin-Burns N., Smith B. J., Kalen A. L., Goswami P. C., Domann F. E. and Spitz D. R. (2006). Mutation of succinate dehydrogenase subunit C results in increased O_2_^−^, oxidative stress, and genomic instability. *Cancer Res.* 66, 7615-7620. 10.1158/0008-5472.CAN-06-0833 16885361

[DMM044925C45] Strittmatter L., Li Y., Nakatsuka N. J., Calvo S. E., Grabarek Z. and Mootha V. K. (2014). CLYBL is a polymorphic human enzyme with malate synthase and beta-methylmalate synthase activity. *Hum. Mol. Genet.* 23, 2313-2323. 10.1093/hmg/ddt624 24334609 PMC3976331

[DMM044925C46] Timmers H. J., Kozupa A., Eisenhofer G., Raygada M., Adams K. T., Solis D., Lenders J. W. and Pacak K. (2007). Clinical presentations, biochemical phenotypes, and genotype-phenotype correlations in patients with succinate dehydrogenase subunit B-associated pheochromocytomas and paragangliomas. *J. Clin. Endocrinol. Metab.* 92, 779-786. 10.1210/jc.2006-2315 17200167

[DMM044925C47] Timmons L. (2006). Construction of plasmids for RNA interference and in vitro transcription of double-stranded RNA. *Methods Mol. Biol.* 351, 109-117. 10.1385/1-59745-151-7:109 16988429

[DMM044925C48] Tretter L., Patocs A. and Chinopoulos C. (2016). Succinate, an intermediate in metabolism, signal transduction, ROS, hypoxia, and tumorigenesis. *Biochim. Biophys. Acta* 1857, 1086-1101. 10.1016/j.bbabio.2016.03.012 26971832

[DMM044925C49] Tseng P.-L., Wu W.-H., Hu T.-H., Chen C.-W., Cheng H.-C., Li C.-F., Tsai W.-H., Tsai H.-J., Hsieh M.-C., Chuang J.-H. et al. (2018). Decreased succinate dehydrogenase B in human hepatocellular carcinoma accelerates tumor malignancy by inducing the Warburg effect. *Sci. Rep.* 8, 3081 10.1038/s41598-018-21361-6 29449614 PMC5814459

[DMM044925C50] Vander Heiden M. G., Cantley L. C. and Thompson C. B. (2009). Understanding the Warburg effect: the metabolic requirements of cell proliferation. *Science* 324, 1029-1033. 10.1126/science.1160809 19460998 PMC2849637

[DMM044925C51] Wadsworth W. G. and Riddle D. L. (1989). Developmental regulation of energy metabolism in Caenorhabditis elegans. *Dev. Biol.* 132, 167-173. 10.1016/0012-1606(89)90214-5 2917691

[DMM044925C52] Xie H., Hanai J., Ren J.-G., Kats L., Burgess K., Bhargava P., Signoretti S., Billiard J., Duffy K. J., Grant A. et al. (2014). Targeting lactate dehydrogenase--a inhibits tumorigenesis and tumor progression in mouse models of lung cancer and impacts tumor-initiating cells. *Cell Metab.* 19, 795-809. 10.1016/j.cmet.2014.03.003 24726384 PMC4096909

