# Supplementary material for: The SDHB Arg230His mutation causing familial paraganglioma alters glycolysis in a new Caenorhabditis elegans model
Source: Dis Model Mech. 2020 Oct 15;13(10):dmm044925. doi: 10.1242/dmm.044925 (PMC7578352; doi:10.1242/dmm.044925)
Supplement: Supplementary information [file dmm-13-044925-s1.pdf]

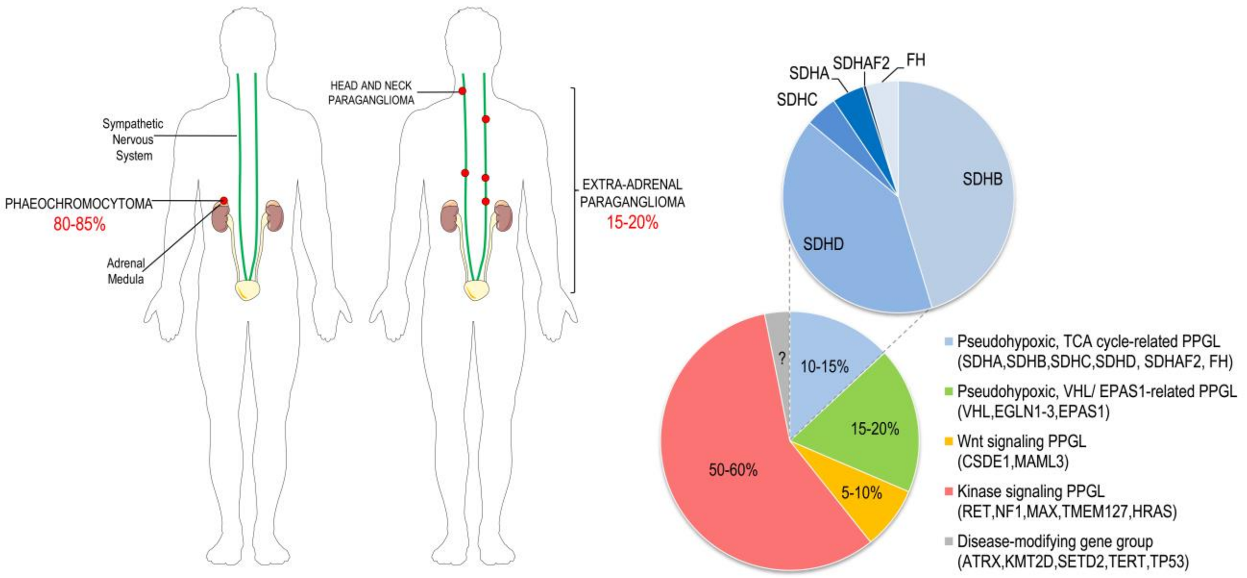

Figure S1. PPGLs (pheochromocytomas/paragangliomas) are neuroendocrine tumors derived from the adrenal gland (pheochromocytoma, 80–85% of PPGLs) and extraadrenal sympathetic and parasympathetic paraganglia (paraganglioma). Sympathetic paragangliomas (including those derived from the adrenal medulla) often secrete catecholamines. 10% of pheochromocytomas and 15-35% of paragangliomas are malignant (Harari and Inabnet, 2011). Genes whose mutations predispose to PPGLs are classified into the following groups: 1. Pseudohypoxic group (TCA cycle-related and VHL/EPAS1-related genes) 2. Wnt signaling group 3. Kinase signaling group 4. Disease-modifying genes. Numbers on the lower circle indicate the distribution of the corresponding mutations in PPGL patients. The majority of cases in the TCA cycle-related PPGLs are caused by mutations in SDH subunit genes (upper circle) (Kantorovich and Pacak, 2018).

Disease Models & Mechanisms • Supplementary information

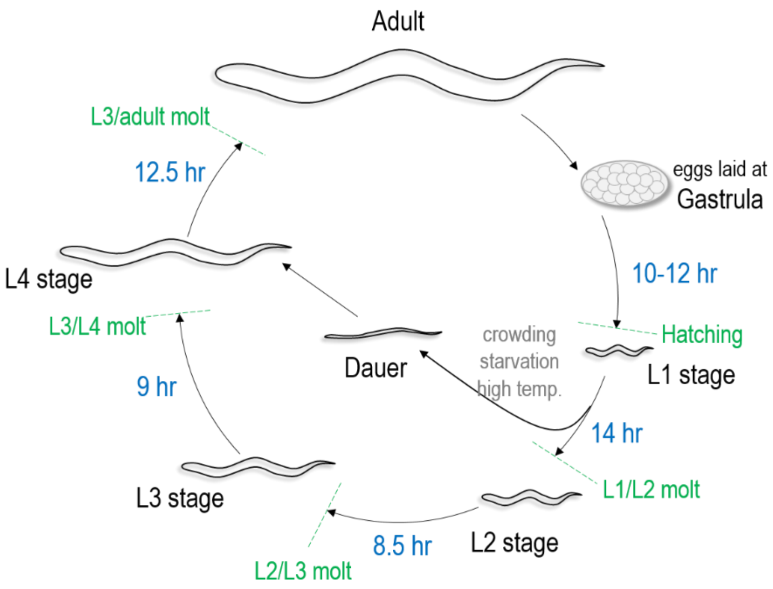

Figure S2. *C. elegans* embryos, after hatching out of the eggshell, develop into adulthood through four larval stages (L1-L4). If conditions are unfavorable (crowding, starvation, high temperature), L1 larvae enter dauer development, where they are able to survive up to several months. Under advantageous environmental conditions, dauer larvae can re-enter normal development.

Disease Models & Mechanisms • Supplementary information

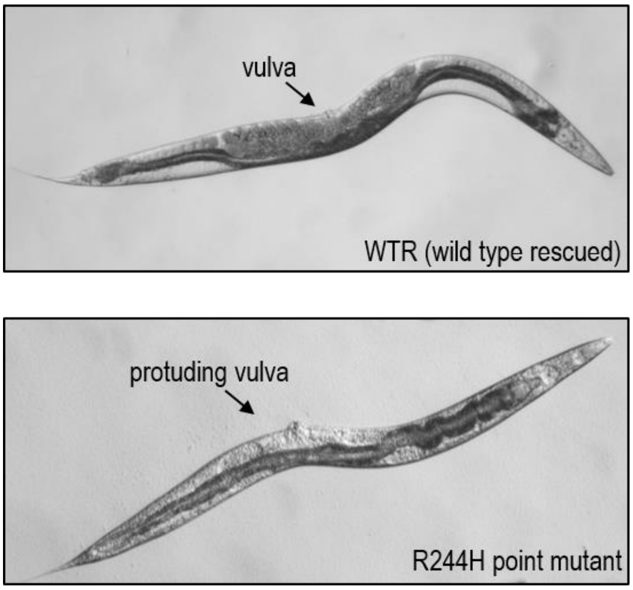

Figure S3. The wild-type *sdhb-1* transgene could efficiently rescue the L2 arrest phenotype of the *gk165* deletional allele, therefore we refer to as wild-type rescued (WTR). However, the G731A point mutant transgene, which corresponds to R244H missense mutation in the protein was not able to complement the deletional mutation. We refer to the G731A point mutants in the deletional background as R244H. DIC images show that WTR animals develop into wild-type, fertile adults, while R244H point mutant worms are sterile adults displaying protruding vulva (Pvl).

Disease Models & Mechanisms • Supplementary information

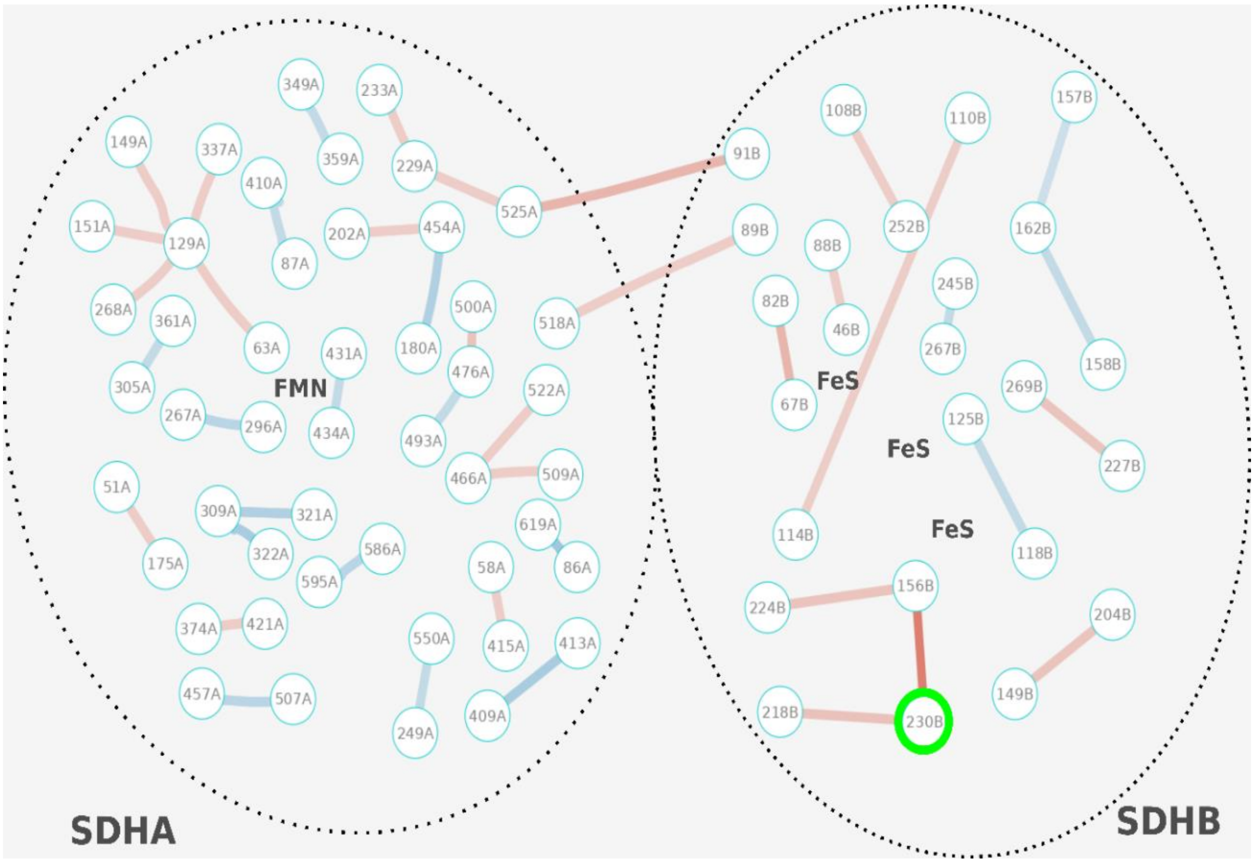

Figure S4. 2-dimensional network graph representation of the side-chain perturbation. The nodes of the graph are residue pairs with contact perturbations greater than 40%. The edges connecting any two nodes indicate that the residue pair was fairly perturbed in the homology models. The extent of perturbation upon mutation is color-coded in RWB space [R=1; W=0; B=-1], with red indicating loss of contact while blue indicating formation of a new contact. The corresponding data is shown in Supplementary Table 7. Due to the 2-dimensional nature of the representation, locations of cofactors and residues were approximated in order to provide a clear depiction. The side-chain perturbation observed in the top 20 homology models upon mutation (highlighted in green) indicates loss of contacts between subunits A and B. A significant perturbation is also found in the vicinity of cofactor FMN of the SDHA.

Disease Models & Mechanisms • Supplementary information

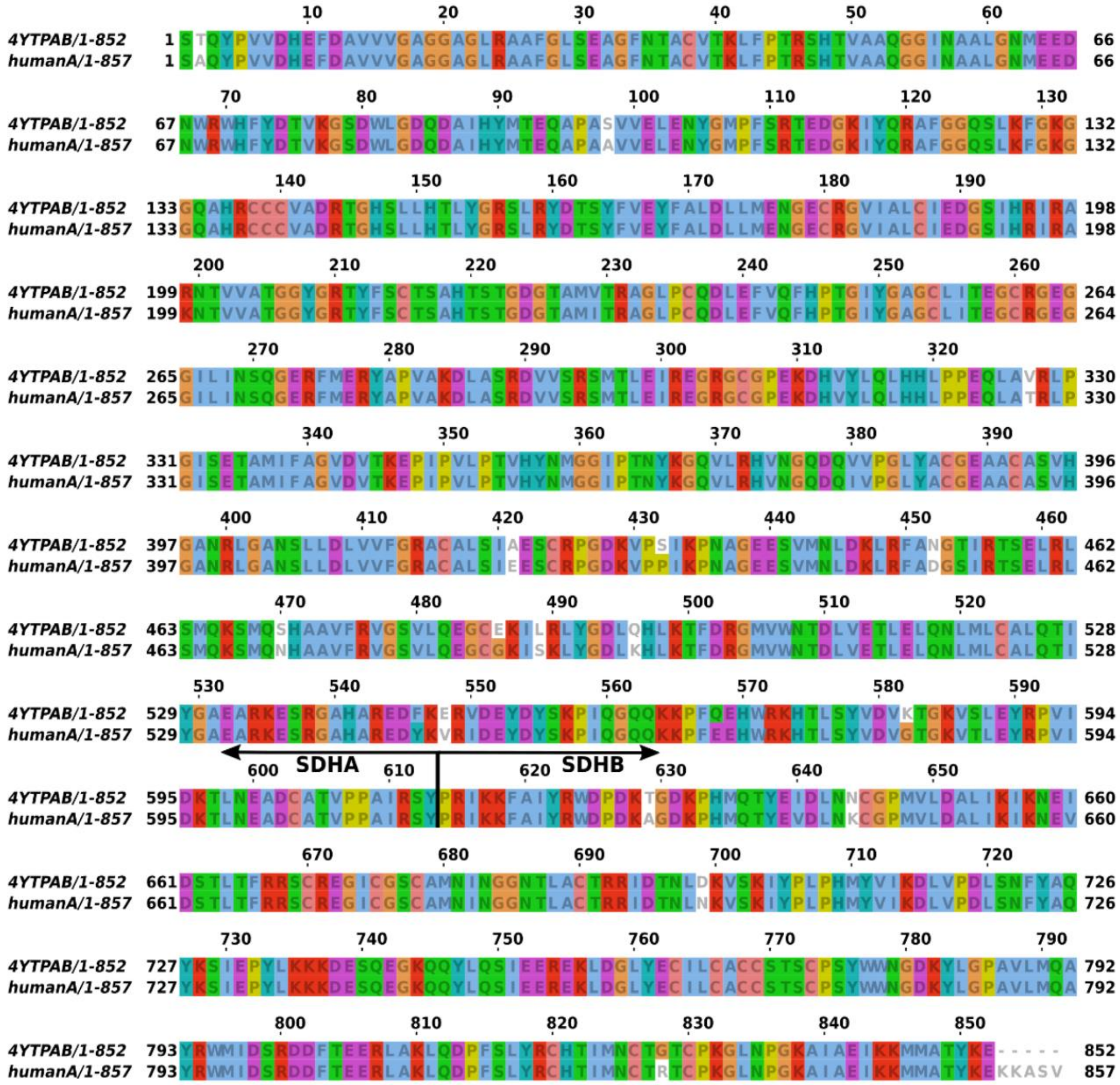

Figure S5. Sequence alignment of subunits A and B of Porcine SDH (PDB id: 4YTP) and human SDH sequenes P31040 (SDHA) and P21912 (SDHB).

Genotype	Number of plates	Number of worms	Censored	Mean lifespan (days)	±SEM (days)	Log Rank P value (with Bonferroni correction)	Independent *t*-test P value (with Bonferroni correction)
**WT**	6	241	2	20.5	0.4		
**WTR**	3	100	1	20.1	0.5		
**R244H**	3	166	3	14.3	0.2	vs. WT P<0.001	vs. WT P<0.001
						vs. WTR P<0.001	vs. WTR P<0.001
sdhb-1 (gk165)	3	125	4	10.5	0.2	vs. WT P<0.001	vs. WT P<0.0001
						vs. R244H P<0.001	vs. R244H P<0.001
**WT; control RNAi**	3	137	3	19.6	0.4		
**WT; *icl-1(RNAi)***	3	119	4	20.8	0.5	vs. control RNAi P=0.018	vs. control RNAi P=0.064
**WTR; control RNAi**	3	142	3	19.1	0.4		
**WTR; *icl-1(RNAi)***	3	130	2	20.0	0.4	vs. control RNAi P=0.031	vs. control RNAi P=0.117
**R244H; control RNAi**	3	155	2	14.8	0.2		
**R244H; *icl-1(RNAi)***	3	155	1	15.2	0.2	vs. control RNAi P=0.581	vs. control RNAi P=0.168
***sdhb-1 (gk165)*; control RNAi**	3	148	6	10.1	0.3		
sdhb-1 (gk165)*; *icl-1(RNAi)	3	188	1	7.9	0.3	vs. control RNAi P<0.001	vs. control RNAi P<0.001

Table S1. Statistics for lifespan data generated in this study. "WT" denotes wild-type; "WTR" indicates the wild-type rescued transgenic strain and "R244H" indicates Arg244His point mutant transgenic animals. *gk165* is a null allele of *sdhb-1*.

Disease Models & Mechanisms • Supplementary information

		**WT** (L2)	**WT** (L3)	***sdhb-1 (gk165)*** (L2)	**WT** (L4)	**WTR** (L4)	**R244H** (L4)
**aKG**	**Mean**	BDL	BDL	BDL	BDL	BDL	BDL
	**±SEM**						
**CIT**	**Mean**	314	327	306	362	325	311
	**±SEM**	±1	±3	±6	±7	±4	±1
**FUM**	**Mean**	16	12	4	228	186	68
	**±SEM**	±1	±2	±2	±33	±4	±3
**LAC**	**Mean**	989	1147	998	2632	2012	4590
	**±SEM**	±31	±108	±179	±333	±360	±41
**MAL**	**Mean**	2147	2151	1940	3338	2731	2230
	**±SEM**	±31	±14	±14	±129	±54	±37
**PYR**	**Mean**	65	97	51	256	202	400
	**±SEM**	±2	±6	±1	±24	±12	±9
**SUC**	**Mean**	25	24	394	738	711	4768
	**±SEM**	±1	±4	±47	±124	±73	±25
**ASP**	**Mean**	BDL	BDL	BDL	174	134	BDL
	**±SEM**				±32	±7	
**GLU**	**Mean**	628	742	531	4858	5220	1391
	**±SEM**	±14	±67	±53	±707	±145	±55

Table S2. Concentrations of metabolites in *sdhb-1(gk165)* deletional, R244H (Arg244His point mutant) animals and their controls (WT (wild-type) and WTR (wild-type rescued) animals) as determined by LC-MS (pmol/million cells; n=6). Abbreviations: alpha-ketoglutarate (aKG); citrate (CIT); fumarate (FUM); lactate (LAC); malate (MAL); pyruvate (PYR); succinate (SUC); aspartate (ASP); glutamate (GLU); below detection limit (BDL); L2 larval stage (L2); L3 larval stage (L3); L4 larval stage (L4).

Disease Models & Mechanisms • Supplementary information

Metabolite ratios	Strains	Mean	±S.E.M.	one-way ANOVA
**succinate-to-fumarate**	WT (L2)	1.5	0.3	
	WT (L3)	2.1	0.0	
	sdhb-1(gk165) (L2)	75.4	14.2	vs. WT (L2) P<0.001<br>vs. WT (L3) P<0.001
	WT (L4)	2.1	0.4	
	WTR (L4)	3.4	0.5	
	R244H (L4)	69.1	4.9	vs. WT (L4) P<0.001<br>vs. WTR (L4) P<0.001
**lactate-to-citrate**	WT (L2)	3.2	0.1	
	WT (L3)	3.1	0.4	
	sdhb-1(gk165) (L2)	4.0	0.8	vs. WT (L2) P=1.00<br>vs. WT (L3) P=1.00
	WT (L4)	4.3	0.9	
	WTR (L4)	5.0	1.4	
	R244H (L4)	14.7	0.2	vs. WT (L4) P<0.001<br>vs. WTR (L4) P<0.001
**pyruvate-to-citrate**	WT (L2)	0.2	0.0	
	WT (L3)	0.3	0.0	
	sdhb-1(gk165) (L2)	0.2	0.0	vs. WT (L2) P=1.00<br>vs. WT (L3) P=1.00
	WT (L4)	0.5	0.1	
	WTR (L4)	0.6	0.1	
	R244H (L4)	1.2	0.0	vs. WT (L4) P<0.001<br>vs. WTR (L4) P<0.001

Table S3. Statistics of metabolite ratios in *sdhb-1(gk165)* deletional, R244H (Arg244His point mutant) animals and their controls (WT (wild-type) and WTR (wild-type rescued) animals). Abbreviations: L2 larval stage (L2); L3 larval stage (L3); L4 larval stage (L4).

Disease Models & Mechanisms • Supplementary information

	Strains	Mean	±S.E.M.	one-way ANOVA
**Basal Respiration**	WT (L2)	61.1	1.2	
	sdhb-1(gk165) (L2)	56.5	1.3	vs. WT (L2) P=1.00
	WT (L4)	72.2	6.0	
	WTR (L4)	67.8	6.5	
	R244H (L4)	67.8	2.8	vs. WT (L4) P=1.00<br>vs. WTR (L4) P=1.00
**Maximal Respiration**	WT (L2)	101.9	9.0	
	sdhb-1(gk165) (L2)	27.0	3.1	vs. WT (L2) P<0.001
	WT (L4)	131.7	2.3	
	WTR (L4)	137.9	8.1	
	R244H (L4)	50.7	3.8	vs. WT (L4) P<0.001<br>vs. WTR (L4) P<0.001
**Spare Capacity**	WT (L2)	40.9	8.4	
	sdhb-1(gk165) (L2)	-29.5	2.3	vs. WT (L2) P<0.001
	WT (L4)	59.5	6.7	
	WTR (L4)	70.1	13.6	
	R244H (L4)	-17.1	3.6	vs. WT (L4) P<0.001<br>vs. WTR (L4) P<0.001
**Non-Mitochondrial Respiration**	WT (L2)	23.1	0.3	
	sdhb-1(gk165) (L2)	12.5	0.4	vs. WT (L2) P<0.001
	WT (L4)	25.7	0.7	
	WTR (L4)	23.3	0.6	
	R244H (L4)	15.0	0.5	vs. WT (L4) P<0.001<br>vs. WTR (L4) P<0.001

Table S4. Statistics for Seahorse data. "WT" denotes wild-type; "WTR" and "R244H" indicate the wild-type rescued transgenic strain and Arg244His point mutant transgenic animals, respectively. *gk165* is a null allele of the *sdhb-1* gene. Abbreviations: L2 larval stage (L2); L3 larval stage (L3); L4 larval stage (L4).

Disease Models & Mechanisms • Supplementary information

	Strains	Mean	±S.E.M.	Independent *t*-test P value
Mitochondrial content	WT (L2)	2.0	0.5	
	sdhb-1(gk165) (L2)	1.2	0.3	vs. WT (L2) P<0.001
	WT (L4)	4.0	0.8	
	WTR (L4)	3.9	0.6	
	R244H (L4)	3.4	0.5	vs. WT (L4) P=0.050<br>vs. WTR (L4) P=0.038

Table S5. Mitochondrial content in *sdhb-1(gk165)* deletional, R244H (Arg244His point mutant) animals and their controls (WT (wild-type N2) and WTR (wild-type rescued) animals). Abbreviations: L2 larval stage (L2); L3 larval stage (L3); L4 larval stage (L4).

	Strains	Mean	±S.E.M.	Independent *t*-test P value
ATP content	WT (L2)	11390858	1654917	
	sdhb-1(gk165) (L2)	5929160	1179916	vs. WT (L2) P=0.010
	WT (L4)	22445048	2895084	
	WTR (L4)	21694782	2681622	
	R244H (L4)	8508257	2995046	vs. WT (L4) P=0.004<br>vs. WTR (L4) P=0.005

Table S6. ATP content in *sdhb-1(gk165)* deletional, R244H (Arg244His point mutant) animals and their controls (WT (wild-type N2) and WTR (wild-type rescued) animals). Abbreviations: L2 larval stage (L2); L3 larval stage (L3); L4 larval stage (L4).

Disease Models & Mechanisms • Supplementary information

Residue1	Residue2	Contact frequency		Residue1	Residue2	Contact frequency
63A	129A	0.4		167A	155B (127B)	-0.4
51A	175A	0.4		457A	507A	-0.45
129A	149A	0.4		476A	493A	-0.4
129A	151A	0.4		466A	509A	0.4
129A	268A	0.4		476A	500A	0.45
229A	233A	0.4		466A	522A	0.4
129A	337A	0.4		586A	595A	-0.45
58A	415A	0.4		518A	89B (61B)	0.4
87A	410A	-0.4		525A	91B (63B)	0.45
267A	296A	-0.45		46B (18B)	88B (60B)	0.4
309A	321A	-0.4		67B (39B)	82B (54B)	0.45
309A	322A	-0.45		110B (39B)	114B (86B)	0.45
180A	454A	-0.4		118B (90B)	125B (97B)	-0.4
202A	454A	0.4		157B (129B)	162B (134B)	-0.4
305A	361A	-0.4		158B (130B)	162B (134B)	-0.4
86A	619A	-0.5		149B (121B)	204B (176B)	0.45
349A	359A	-0.4		108B (80B)	252B (224B)	0.4
229A	525A	0.4		156B (128B)	224B (196B)	0.45
374A	421A	0.4		156B (128B)	230B (202B)	1
249A	550A	-0.4		218B (190B)	230B (202B)	0.45
409A	413A	-0.45		227B (199B)	269B (241B)	0.45
431A	434A	-0.4		245B (217B)	267B (239B)	-0.4

Table S7. Differences between wildtype and mutant of averaged residue-pair contact frequencies from the twenty top scoring homology models. This table lists the residue pairs that exhibit a loss (positive) or gain (negative) of contacts in more than 40% (> 8 out of the 20 models) going from wildtype to the R230H mutant, as shown in Figure 6C, annotated in Figure 6D, and additionally depicted as a 2-dimensional network graph in Supplementary Figure 4. Residue 1 and residue 2 form residue pairs. In the residue notation *XY*, *X* refers to residue number based on human numbering and *Y* refers to the protein chain. The template *Sus scrofa* residue numbering for SDHB corresponding to PDB id: 4YTP is indicated in parenthesis.

Disease Models & Mechanisms • Supplementary information

Gene	Mutant strain	Control strain	Fold change mean	Fold change ±S.E.M.	two-tail Student t-test (with Bonferroni correction)
cts-1	*sdhb-1 (gk165)*	WT L2	1.1	0.0	P=0.315
		WT L3	1.0	0.1	P=0.996
	R244H	WT L4	1.1	0.0	P=0.006
		WTR L4	1.1	0.0	P=0.001
aco-2	*sdhb-1 (gk165)*	WT L2	1.2	0.1	P=0.023
		WT L3	1.1	0.0	P=0.119
	R244H	WT L4	1.1	0.0	P=0.003
		WTR L4	1.2	0.1	P=0.007
idh-1	*sdhb-1 (gk165)*	WT L2	1.0	0.0	P=0.296
		WT L3	0.8	0.1	P=0.104
	R244H	WT L4	1.1	0.0	P=0.013
		WTR L4	1.1	0.0	P=0.038
idh-2	*sdhb-1 (gk165)*	WT L2	0.8	0.2	P=0.248
		WT L3	0.8	0.2	P=0.307
	R244H	WT L4	1.2	0.1	P=0.069
		WTR L4	1.4	0.2	P=0.030
ogdh-1	*sdhb-1 (gk165)*	WT L2	0.8	0.1	P=0.150
		WT L3	0.7	0.1	P=0.034
	R244H	WT L4	1.1	0.0	P=0.004
		WTR L4	1.2	0.0	P=0.006
suca-1	*sdhb-1 (gk165)*	WT L2	0.7	0.1	P=0.014
		WT L3	0.5	0.1	P=0.001
	R244H	WT L4	1.0	0.0	P=0.170
		WTR L4	1.0	0.0	P=0.440
sucg-1	*sdhb-1 (gk165)*	WT L2	0.7	0.2	P=0.119
		WT L3	0.7	0.2	P=0.118
	R244H	WT L4	0.6	0.1	P=0.005
		WTR L4	0.6	0.1	P=0.011
sucl-1	*sdhb-1 (gk165)*	WT L2	0.7	0.1	P=0.004
		WT L3	0.7	0.1	P=0.002
	R244H	WT L4	1.1	0.0	P=0.007
		WTR L4	1.1	0.0	P=0.006
sucl-2	*sdhb-1 (gk165)*	WT L2	0.6	0.3	P=0.265
		WT L3	0.4	0.1	P=0.080
	R244H	WT L4	1.0	0.0	P=0.838
		WTR L4	1.0	0.0	P=0.664
sdha-1	*sdhb-1 (gk165)*	WT L2	0.7	0.1	P=0.028
		WT L3	0.7	0.1	P=0.020
	R244H	WT L4	1.1	0.0	P=0.015
		WTR L4	1.2	0.0	P=0.001
sdhb-1	*sdhb-1*	WT L2	0.4	0.1	P=0.010

Disease Models & Mechanisms • Supplementary information

	(gk165)	WT L3	0.3	0.1	P=0.001
	R244H	WT L4	0.9	0.1	P=0.300
		WTR L4	1.1	0.1	P=0.262
mev-1	*sdhb-1 (gk165)*	WT L2	1.1	0.1	P=0.467
		WT L3	1.0	0.1	P=0.995
	R244H	WT L4	1.1	0.0	P=0.066
		WTR L4	1.0	0.0	P=0.321
sdhd-1	*sdhb-1 (gk165)*	WT L2	0.7	0.2	P=0.300
		WT L3	0.7	0.3	P=0.376
	R244H	WT L4	1.0	0.0	P=0.655
		WTR L4	0.9	0.0	P=0.256
fum-1	*sdhb-1 (gk165)*	WT L2	0.8	0.2	P=0.318
		WT L3	0.7	0.2	P=0.277
	R244H	WT L4	1.1	0.0	P=0.022
		WTR L4	1.1	0.0	P=0.016
mdh-1	*sdhb-1 (gk165)*	WT L2	1.1	0.1	P=0.411
		WT L3	1.0	0.1	P=0.931
	R244H	WT L4	1.2	0.0	P=0.004
		WTR L4	1.2	0.0	P=0.060
mdh-2	*sdhb-1 (gk165)*	WT L2	0.8	0.1	P=0.067
		WT L3	0.7	0.1	P=0.032
	R244H	WT L4	1.1	0.0	P=0.066
		WTR L4	1.1	0.0	P=0.022
icl-1	*sdhb-1 (gk165)*	WT L2	1.3	0.0	P=0.038
		WT L3	1.2	0.1	P=0.114
	R244H	WT L4	1.4	0.1	P<0.001
		WTR L4	1.4	0.1	P=0.001
pyk-1	*sdhb-1 (gk165)*	WT L2	1.1	0.3	P=0.456
		WT L3	0.5	0.1	P=0.000
	R244H	WT L4	1.0	0.0	P=0.600
		WTR L4	1.1	0.1	P=0.138
pdha-1	*sdhb-1 (gk165)*	WT L2	1.1	0.1	P=0.333
		WT L3	1.0	0.1	P=0.908
	R244H	WT L4	1.1	0.0	P=0.006
		WTR L4	1.1	0.0	P=0.020
pdhb-1	*sdhb-1 (gk165)*	WT L2	1.0	0.0	P=0.964
		WT L3	0.9	0.0	P=0.029
	R244H	WT L4	1.0	0.0	P=0.895
		WTR L4	1.0	0.0	P=0.001
pck-1	*sdhb-1 (gk165)*	WT L2	1.0	0.1	P=0.590
		WT L3	0.9	0.1	P=0.206
	R244H	WT L4	1.2	0.0	P=0.021
		WTR L4	1.3	0.1	P=0.048
pck-2	*sdhb-1 (gk165)*	WT L2	1.0	0.2	P=0.500
		WT L3	0.9	0.2	P=0.639
	R244H	WT L4	1.3	0.0	P<0.001

		WTR L4	1.3	0.1	P<0.001
pyc-1	*sdhb-1 (gk165)*	WT L2	1.4	0.3	P=0.471
		WT L3	0.9	0.1	P=0.356
	R244H	WT L4	1.2	0.0	P<0.001
		WTR L4	1.4	0.1	P<0.001
men-1	*sdhb-1 (gk165)*	WT L2	1.0	0.2	P=0.389
		WT L3	0.8	0.1	P=0.037
	R244H	WT L4	0.9	0.0	P=0.081
		WTR L4	1.0	0.0	P=0.294
ldh-1	*sdhb-1 (gk165)*	WT L2	1.1	0.2	P=0.822
		WT L3	1.0	0.1	P=0.648
	R244H	WT L4	1.3	0.1	P=0.055
		WTR L4	1.3	0.1	P=0.043
gdh-1	*sdhb-1 (gk165)*	WT L2	1.0	0.0	P=0.542
		WT L3	0.9	0.0	P=0.020
	R244H	WT L4	1.0	0.0	P=0.289
		WTR L4	1.0	0.0	P=0.329
got-2.2	*sdhb-1 (gk165)*	WT L2	1.2	0.1	P=0.148
		WT L3	1.0	0.0	P=0.646
	R244H	WT L4	1.2	0.0	P=0.026
		WTR L4	1.2	0.1	P=0.129

Table S8: Statistics for RNA-Seq data. "WT" denotes wild-type; "WTR" and "R244H" indicate the wild-type rescued transgenic strain and Arg244His point mutant transgenic animals, respectively. *gk165* is a null allele of the *sdhb-1* gene. Abbreviations: L2 larval stage (L2); L3 larval stage (L3); L4 larval stage (L4). Genes encoding the following enzymes were analyzed: CTS-1: citrate synthase, ACO-2: aconitase, IDH-1/-2: isocitrate dehydrogenase, OGDH-1: oxoglutarate dehydrogenase, SUCA-1: succinyl-CoA ligase (ATP-specific) beta subunit, SUCG-1: succinyl-CoA ligase (GTP-specific) beta subunit, SUCL-1: succinyl-CoA ligase (ATP/GTP-specific) alpha subunit, SUCL-2: succinyl-CoA ligase (ATP/GTP-specific) alpha subunit, SDHA-1: succinate dehydrogenase A subunit, SDHB-1: succinate dehydrogenase B subunit, MEV-1: succinate dehydrogenase C subunit, SDHD-1: succinate dehydrogenase D subunit, FUM-1: fumarase, MDH-1/-2: malate dehydrogenase, ICL-1: isocitrate lyase/ malate dehydrogenase, PYK-1: pyruvate kinase, PDHA-1: pyruvate dehydrogenase alpha subunit, PDHB-1: pyruvate dehydrogenase beta subunit, PCK-1/-2: phosphoenolpyruvate carboxykinase, PYC-1: pyruvate carboxylase, MEN-1: malic enzyme, LDH-1: lactate dehydrogenase, GDH-1: glutamate dehydrogenase and GOT-2.2: glutamate oxoacetate transaminase.

Disease Models & Mechanisms • Supplementary information

Gene	Mutant strain	Control strain	Fold change mean	Fold change ±S.E.M.	one-way ANOVA
aco-2	*sdhb-1 (gk165)*	WT L2	3.7	0.5	P<0.001
		WT L3	1.7	0.3	P=0.134
	R244H	WT L4	2.8	0.1	P<0.001
		WTR L4	3.2	0.2	P<0.001
fum-1	*sdhb-1 (gk165)*	WT L2	1.8	0.1	P<0.001
		WT L3	1.2	0.1	P=0.528
	R244H	WT L4	2.2	0.2	P<0.001
		WTR L4	2.6	0.2	P<0.001
gdh-1	*sdhb-1 (gk165)*	WT L2	1.5	0.1	P=0.261
		WT L3	0.7	0.0	P=0.445
	R244H	WT L4	1.8	0.2	P=0.016
		WTR L4	1.5	0.1	P=0.231
icl-1	*sdhb-1 (gk165)*	WT L2	21.3	2.6	P<0.001
		WT L3	20.4	2.4	P<0.001
	R244H	WT L4	11.0	1.5	P<0.001
		WTR L4	10.7	1.0	P<0.001
idh-2	*sdhb-1 (gk165)*	WT L2	1.4	0.2	P=0.875
		WT L3	0.9	0.1	P=1.000
	R244H	WT L4	2.1	0.3	P<0.001
		WTR L4	3.2	0.4	P<0.001
ldh-1	*sdhb-1 (gk165)*	WT L2	1.6	0.1	P<0.001
		WT L3	1.2	0.1	P=0.192
	R244H	WT L4	3.3	0.2	P<0.001
		WTR L4	4.3	0.5	P<0.001
men-1	*sdhb-1 (gk165)*	WT L2	1.4	0.1	P=0.473
		WT L3	1.0	0.1	P=1.000
	R244H	WT L4	1.4	0.1	P=0.612
		WTR L4	1.5	0.1	P=0.181
pck-2	*sdhb-1 (gk165)*	WT L2	6.0	0.5	P<0.001
		WT L3	2.8	0.4	P<0.001
	R244H	WT L4	6.3	0.5	P<0.001
		WTR L4	7.0	0.5	P<0.001
pyc-1	*sdhb-1 (gk165)*	WT L2	3.7	0.3	P<0.001
		WT L3	2.7	0.4	P<0.001
	R244H	WT L4	4.0	0.3	P<0.001
		WTR L4	3.6	0.2	P<0.001
sdha-1	*sdhb-1 (gk165)*	WT L2	1.6	0.2	P=0.652
		WT L3	1.1	0.1	P=1.000
	R244H	WT L4	2.5	0.4	P<0.001
		WTR L4	3.3	0.3	P<0.001

Disease Models & Mechanisms • Supplementary information

Table S9: Statistics for RT-qPCR data. "WT" denotes wild-type; "WTR" and "R244H" indicate the wild-type rescued transgenic strain and Arg244His point mutant transgenic animals, respectively. *gk165* is a null allele of the *sdhb-1* gene. Abbreviations: L2 larval stage (L2); L3 larval stage (L3); L4 larval stage (L4). Genes encoding the following enzymes were analyzed: ACO-2: aconitase, FUM-1: fumarase, GDH-1: glutamate dehydrogenase, ICL-1: isocitrate lyase/ malate dehydrogenase, IDH-2: isocitrate dehydrogenase, LDH-1: lactate dehydrogenase, MEN-1: malic enzyme, PCK-2: phosphoenolpyruvate carboxykinase, PYC-1: pyruvate carboxylase and SDHA-1: succinate dehydrogenase A subunit.

Disease Models & Mechanisms • Supplementary information

Genotype	Number of plates	Number of worms	Mean (%)	±SEM	one-way ANOVA
**WT; control RNAi**	3	1063	0.1	0.2	
**WT; *icl-1(RNAi)***	3	959	0.1	0.2	vs. control RNAi P=1.000
**WTR; control RNAi**	3	1138	0.1	0.2	
**WTR; *icl-1(RNAi)***	3	1198	0.2	0.1	vs. control RNAi P=1.000
**R244H; control RNAi**	3	1415	0.1	0.1	
**R244H; *icl-1(RNAi)***	3	1380	1.3	0.8	vs. control RNAi P=1.000
***sdhb-1 (gk165)*; control RNAi**	3	1303	4.8	3.4	
sdhb-1 (gk165)*; *icl-1(RNAi)	3	1150	13.2	2.9	vs. control RNAi P<0.001

Table S10: Statistics to analyze effects of *icl-1(RNAi)* exerted on viability of *sdhb-1* mutant and wild-type embryos. "WT" denotes wild-type; "WTR" and "R244H" indicate the wild-type rescued transgenic strain and Arg244His point mutant transgenic animals, respectively. *gk165* is a null allele of the *sdhb-1* gene. Abbreviations: L2 larval stage (L2); L3 larval stage (L3); L4 larval stage (L4).

Disease Models & Mechanisms • Supplementary information

Genotype	Final concentration of LDH inhibitor (GSK 2837808A)	Number of plates	Number of worms	Mean (%)	±SEM	one-way ANOVA
**R244H**	0 μM (control)	5	237	1.0	1.4	
	1 μM	3	136	1.1	1.3	vs. control P=1.000
	10 μM	6	264	12.4	4.8	vs. control P<0.001

Table S11: Treatment of R244H point mutants by LDH-A inhibitor GSK2837808A results in L2/L3 arrest. Point mutants were treated by the compound at 1 and 10 μM concentrations. The compound significantly affected the development of mutants only at 10 μM concentration compared to untreated animals.

**Oligonucleotides for *psdhb-1::gfp* construct**	
Primer: *psdhb-1*	Forward: GCGGGCGCGCCGTTTCGCTAATTTTGAACAATAGAAG<br>Reverse: CGCGCGGCCGCGGGCCAACATCTGAGATTTATTC
**Oligonucleotides for RT-qPCR measurements**	
Primer: *aco-2*	Forward: CCCGTGCTAAGGACCTCAAC<br>Reverse: GTTCCGATGAGAAGAAGTCCTGG
Primer: *fum-1*	Forward: TCGAGTTGAATGTTTTCAAGCCAC<br>Reverse: ATAAGTGATTCACGCATAATCTTGGC
Primer: *gdh-1*	Forward: CAGTTAACAAGGTCCTTTACATCACC<br>Reverse: TGACCTCATCTTCGCACACATC
Primer: *icl-1*	Forward: TTCGAGTTGATGAAGGCCTATATCG<br>Reverse: CAAACGAGACGCATTCAAGTGAC
Primer: *idh-2*	Forward: TTGTTGCTCAAGGATACGGATCG<br>Reverse: GGTTTGTAGACGTCGAGTTACCC
Primer: *ldh-1*	Forward: AGGCTGATACTGACTACTCTATCACC<br>Reverse: GTACTTGACAAGTTGTGGGATGATTC
Primer: *men-1*	Forward: ATGATATTCAAGGTACCGCCGC<br>Reverse: GTCCATCATCTGTCGGACACAC
Primer: *pck-2*	Forward: GCTCATCCAAACTCGCGTTTC<br>Reverse: TCCCATGAGTTAGTCTCATAAATGAGTG
Primer: *pyc-1*	Forward: GAATTCAACAAGGTTATGGTGGCC<br>Reverse: ATAGGCTTCGTCAGCTTTCAGAC
Primer: *sdha-1*	Forward: AGCTCAAGGAGGAATCAATGCTG<br>Reverse: TCAAGCTCAATGACAGCTCTCTC
Primer: *cdc-42*	Forward: GACAATTACGCCGTCACAGTAATG<br>Reverse: TGAAGCTGGAGCAACCACG
**Oligonucleotides for RNAi construct**	
Primer: *icl-1 (gei-7)*	Forward: TATGAGCTCTTATCAGGTAGTCAAATCGGCTC<br>Reverse: ATAGGTACCAGGAACTCCGCAGACATCAG

Table S12: List of primers used in this study.

Disease Models & Mechanisms • Supplementary information

Supplementary references

Harari, A. and Inabnet, W. B., 3rd (2011) 'Malignant pheochromocytoma: a review', *Am J Surg* 201(5): 700-8.

Kantorovich, V. and Pacak, K. (2018) 'New insights on the pathogenesis of paraganglioma and pheochromocytoma', *F1000Res* 7.

Disease Models & Mechanisms • Supplementary information
